# Author Correction: Partial inhibition of mitochondrial complex I ameliorates Alzheimer’s disease pathology and cognition in APP/PS1 female mice

**DOI:** 10.1038/s42003-024-05810-9

**Published:** 2024-02-26

**Authors:** Andrea Stojakovic, Sergey Trushin, Anthony Sheu, Layla Khalili, Su-Youne Chang, Xing Li, Trace Christensen, Jeffrey L. Salisbury, Rachel E. Geroux, Benjamin Gateno, Padraig J. Flannery, Mrunal Dehankar, Cory C. Funk, Jordan Wilkins, Anna Stepanova, Tara O’Hagan, Alexander Galkin, Jarred Nesbitt, Xiujuan Zhu, Utkarsh Tripathi, Slobodan Macura, Tamar Tchkonia, Tamar Pirtskhalava, James L. Kirkland, Rachel A. Kudgus, Renee A. Schoon, Joel M. Reid, Yu Yamazaki, Takahisa Kanekiyo, Song Zhang, Emirhan Nemutlu, Petras Dzeja, Adam Jaspersen, Ye In Christopher Kwon, Michael K. Lee, Eugenia Trushina

**Affiliations:** 1 Department of Neurology, Mayo Clinic, 200 First St. SW, Rochester, MN 55905 USA; 2 Institute for Translational Neuroscience, University of Minnesota Twin Cities, 2101 6th Street SE, Minneapolis, MN 55455 USA; 3 Department of Neurologic Surgery, Mayo Clinic, 200 First St. SW, Rochester, MN 55905 USA; 4 Department of Physiology and Biomedical Engineering, Mayo Clinic, 200 First St. SW, Rochester, MN 55905 USA; 5 Division of Biomedical Statistics and Informatics, Department of Health Sciences Research, Mayo Clinic, 200 First St. SW, Rochester, MN 55905 USA; 6 Microscopy and Cell Analysis Core, Mayo Clinic, 200 First St. SW, Rochester, MN 55905 USA; 7 Department of Biochemistry and Molecular Biology, Mayo Clinic, 200 First St. SW, Rochester, MN 55905 USA; 8 Institute for Systems Biology, Seattle, WA 98109-5263 USA; 9 Division of Neonatology, Department of Pediatrics, Columbia University, 116th St & Broadway, New York, NY 10027 USA; 10 Robert and Arlene Kogod Center on Aging, Mayo Clinic, 200 First St. SW, Rochester, MN 55905 USA; 11 Department of Molecular Pharmacology and Experimental Therapeutics, Mayo Clinic, 200 First St. SW, Rochester, MN 55905 USA; 12 Department of Neuroscience, Mayo Clinic, 4500 San Pablo Road, Jacksonville, FL 32224 USA; 13 Department of Cardiovascular Medicine, Mayo Clinic, 200 First St. SW, Rochester, MN 55905 USA; 14 Faculty of Pharmacy, Department of Analytical Chemistry, Hacettepe University, Sihhiye, Ankara, 06100 Turkey

**Keywords:** Alzheimer's disease, Alzheimer's disease

Correction to: *Communications Biology* **4**: 61; 10.1038/s42003-020-01584-y, published online 08 January 2021.

The original version of this article contained several errors in Fig. 3 and Supplementary Fig. 10 related to incomplete disclosure of outliers and how these were treated, and inaccurate reporting of sample sizes in Fig. 3a. Several changes have been made to throughout the main text to reflect these changes, including:

- The legend for Fig. 3
  - The original legend: “**Fig. 3 CP2 treatment increases glucose uptake and utilization in symptomatic APP/PS1 mice. a** Glucose uptake was increased in the brain of CP2- treated APP/PS1 mice measured using FDG-PET after 9 months of treatment. **b** Quantification of glucose uptake by FDG-PET imaging from **a**. NTG, n = 8 mice per group; NTG+ CP2, n = 4 mice per group; APP/PS1, n = 5 mice per group; APP/PS1+ CP2, n = 8 mice per group. **c** Changes in respiratory exchange ratio (RER) recorded in all treatment groups over 44 h during ad lib fed and fasting states. **d** Glucose oxidation was increased in CP2-treated APP/PS1 mice fed ad lib based on CLAMS data from **c**. Gray bars indicate fat consumption; orange bars indicate carbohydrate and protein oxidation. **e** Metabolic flexibility is increased in CP2-treated APP/PS1 mice based on their ability to switch from carbohydrates to fat between feeding and fasting states. **c–e** NTG, n = 16 mice per group; NTG+ CP2, n = 20 mice per group; APP/PS1, n = 15 mice per group; APP/PS1+ CP2, n = 19 mice per group. **f–h** CP2 treatment reduces fasting insulin levels in plasma of APP/PS1 mice **(f)**; increases glucose tolerance in NTG mice measured by intraperitoneal glucose tolerance test (IPGTT) **(g)**; and displays tendency to improve intraperitoneal insulin sensitivity test (IPIST) in NTG and APP/PS1 mice **(h)** after 9–10 months of treatment. n = 5 mice per group. **i** Western blot analysis conducted in the brain tissue of APP/PS1 mice treated with CP2 for 13 months indicates increased IGF-1signaling, expression of Glut 3 and 4 transporters and changes in pyruvate dehydrogenase (PDH) activation associated with glucose utilization in the TCA cycle. **j** Representative 31P NMR spectra with peaks corresponding to energy metabolites, including inorganic phosphate (Pi), phosphocreatine (PCr), and three phosphate group peaks for ATP generated in living NTG and APP/PS1 mice after 9 months of vehicle or CP2 treatment. **k** Phosphocreatine/ATP ratio calculated based on the 31P NMR in vivo spectra from **j**. NTG, n = 4 mice per group; NTG+ CP2, n = 3 mice per group; APP/PS1, n = 6 mice per group; APP/PS1+ CP2, n = 6 mice per group. Data are presented as mean ± S.E.M. Data were analyzed by two-way ANOVA with Fisher’s LSD post hoc test. *P < 0.05, **P < 0.01. In all graphs: APP/PS1, orange line; NTG, black line; NTG+ CP2, blue line; APP/PS1+ CP2, red line.”
  - Has now been corrected to: “**Fig. 3 CP2 treatment increases glucose uptake and utilization in symptomatic APP/PS1 mice. a** Glucose uptake was increased in the brain of CP2-treated APP/PSI mice measured using FDG-PET after 1–2.6 months of treatment in two independent cohorts of mice. Representative images are from one of the cohorts. **b** Quantification of glucose uptake by FDG-PET imaging from **a**. NTG, n = 7 mice per group; NTG+ CP2, n = 4 mice per group; APP/PS1, n = 5 mice per group; APP/PS1+ CP2, n = 10 mice per group. **c** Changes in respiratory exchange rate (RER) recorded in all treatment groups over 44 h during ad lib fed and fasting states in mice treated for 9 months. **d** Glucose oxidation was increased in CP2-treated APP/PS1 mice fed ad lib based on CLAMS data from **c**. Gray bars indicate fat consumption; orange bars indicate carbohydrate and protein oxidation. **e** Metabolic flexibility is increased in CP2-treated APP/PS1 mice based on their ability to switch from carbohydrates to fat between feeding and fasting states. **c–e** NTG, n = 16 mice per group; NTG+ CP2, n = 20 mice per group; APP/PS1, n =15 mice per group; APP/PS1+ CP2, n = 19 mice per group. **f–h** CP2 treatment reduces fasting insulin levels in plasma of APP/PS1 mice **(f)**; increases glucose tolerance in NTG mice measured by intraperitoneal glucose tolerance test (IPGTT) **(g)**; and displays tendency to improve intraperitoneal insulin sensitivity test (IPIST) in NTG and APP/PS1 mice **(h)** after 9–10 months of treatment. n=5 mice per group. The outliers in (**h**) included 1 NTG+CP2 and 2 APP/PS1+CP2 mice that were excluded from the graph. **i** Western blot analysis conducted in the brain tissue of APP/PS1 mice treated with CP2 for 13 months indicates increased IGF-1signaling, expression of Glut 3 and 4 transporters and changes in pyruvate dehydrogenase (PDH) activation associated with glucose utilization in the TCA cycle. **j** Representative 31P NMR spectra with peaks corresponding to energy metabolites, including inorganic phosphate (Pi), phosphocreatine (PCr), and three phosphate group peaks for ATP generated in living NTG and APP/PS1 mice after 9 months of vehicle or CP2 treatment. **k** Phosphocreatine/ATP ratio calculated based on the 31P NMR in vivo spectra from **j**. NTG, n = 4 mice per group; NTG+ CP2, n = 3 mice per group; APP/PS1, n = 6 mice per group; APP/PS1+ CP2, n = 6 mice per group. Data are presented as mean ± S.E.M. Data were analyzed by two-way ANOVA with Fisher’s LSD post hoc test. *P < 0.05, **P < 0.01. In all graphs: APP/PS1, orange line; NTG, black line; NTG+ CP2, blue line; APP/PS1+ CP2, red line.”
- The “Statistics and Reproducibility” section in the Methods.
  - Which originally read as: “The statistical analyses were performed using the GraphPad Prism (Version 8, GraphPad Software, Inc., La Jolla, Ca). Statistical comparisons among four groups concerning behavioral and metabolic tests, immunoreactivity, metabolomics, plasma cytokine panel, body composition, electron microscopy imaging, FDG-PET, 31P NMR, electrophysiology, were analyzed by two-way ANOVA, the two-tailed unpaired and paired Student t test, where appropriate. The Fisher’s LSD post hoc analysis was used if significant interaction among groups was found. A linear regression analysis was applied to determine differences among the groups in body weights and age-related loss of TH+ axons and neurons. Significant differences between vehicle and CP2-treated groups within the same genotype and differences among NTG, APP/PS1, and APP/PS1 +CP2 mice were considered in the final analysis. Data are presented as mean ± S.E.M. for each group of mice. All P values generated in this study are presented in Supplementary Data 20, and individual values used to generate plots in the manuscript are presented in Supplementary Data 21. Sample sizes were determined by setting a minimum n number for in vitro biological replicates at 3, to allow for statistical testing, however in most cases n numbers were higher. All replicates displayed in this paper are biological replicates, technical replicates (usually 3) were performed and used to generate the means for each biological replicate. At the initiation of each experiment, all available data from previous studies were assessed and used to adequately design the experiment to have at least 80% power to detect a biologically meaningful effect size while controlling the type I error rate at 5%. Animal allocation to treatment groups was randomized. We were blinded to both the genetic and treatment information, being unblinded after analysis was complete.”
  - And has now been corrected to: “The statistical analyses were performed using the GraphPad Prism (Version 8, GraphPad Software, Inc., La Jolla, Ca). Statistical comparisons among four groups concerning behavioral and metabolic tests, immunoreactivity, metabolomics, plasma cytokine panel, body composition, electron microscopy imaging, FDG-PET, 31P NMR, electrophysiology, were analyzed by two-way ANOVA, the two-tailed unpaired and paired Student t test, where appropriate. The Fisher’s LSD post hoc analysis was used if significant interaction among groups was found. A linear regression analysis was applied to determine differences among the groups in body weights and age-related loss of TH+ axons and neurons. Significant differences between vehicle and CP2-treated groups within the same genotype and differences among NTG, APP/PS1, and APP/PS1 +CP2 mice were considered in the final analysis. Data are presented as mean ± S.E.M. for each group of mice. All P values generated in this study are presented in Supplementary Data 20, and individual values used to generate plots in the manuscript are presented in Supplementary Data 21. In reporting the outcomes of the IPIST experiments, 1 NGT+CP2 and 2 APP/PS1+CP2 mice were excluded from the graph based on the application of the Grubb’s test to identify the outliers. Sample sizes were determined by setting a minimum n number for in vitro biological replicates at 3, to allow for statistical testing, however in most cases n numbers were higher. All replicates displayed in this paper are biological replicates, technical replicates (usually 3) were performed and used to generate the means for each biological replicate. At the initiation of each experiment, all available data from previous studies were assessed and used to adequately design the experiment to have at least 80% power to detect a biologically meaningful effect size while controlling the type I error rate at 5%. Animal allocation to treatment groups was randomized. We were blinded to both the genetic and treatment information, being unblinded after analysis was complete.”

Supplementary Figs. 10c,e have also been updated to include all data points (including outliers) in the analysis.

- The original Supp Fig. 10:
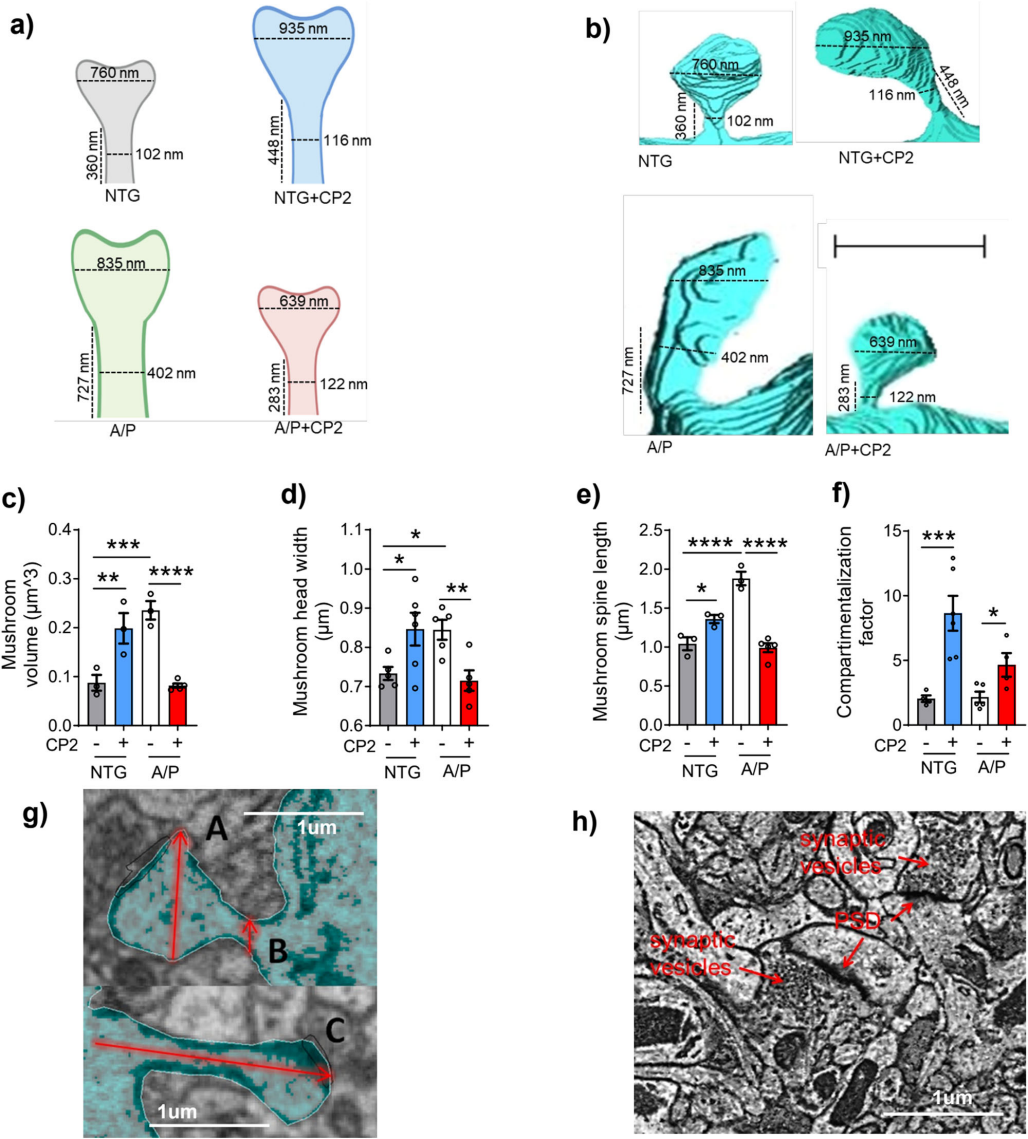
- Has now been corrected:

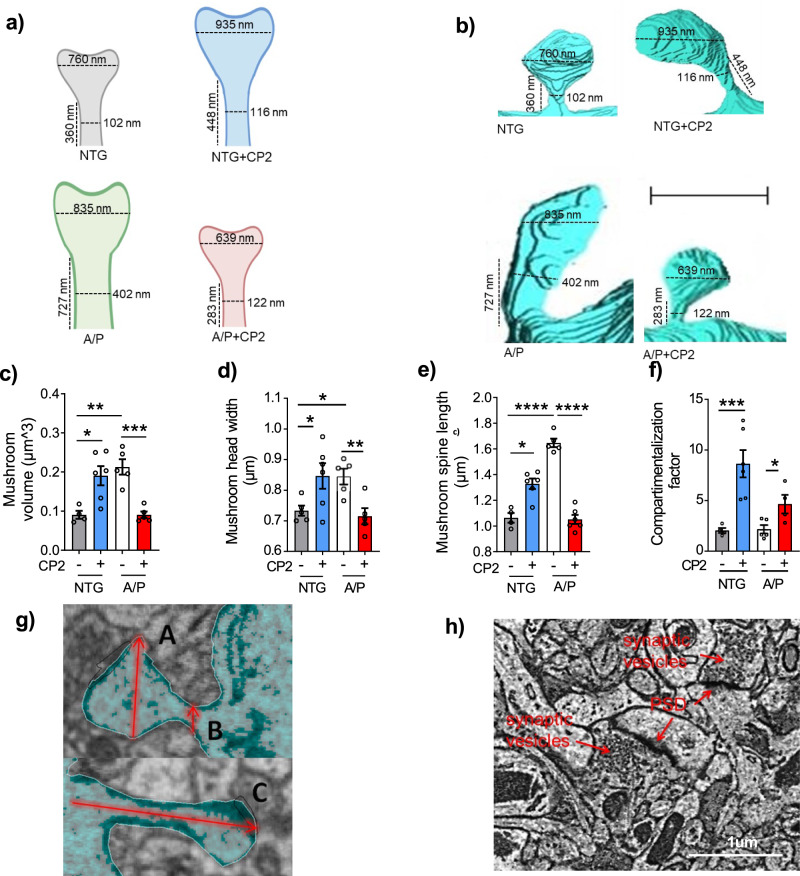
and the legend for Supplementary Fig. 10 was updated to include more details on the methodology.

- The original legend: “**h)** Representative EM micrograph of the CA1 hippocampal region utilized in the calculation of active synapses. Identification of synapses was done based on the presence of postsynaptic density (PSD) and synaptic vesicles. Scale bar, 1 μm.”
- Has now been corrected to: “**h)** Representative EM micrograph of the CA1 hippocampal region utilized in the calculation of active synapses. Identification of synapses was done based on the presence of postsynaptic density (PSD) and synaptic vesicles. Scale bar, 1 μm. Data are presented as mean ± S.E.M. A two-way ANOVA with Fisher`s LSD post hoc test was used for statistical analysis. Reconstructions were done in one mouse per group using 4 dendrites from NTG; 6 dendrites from NTG+CP2; 5 dendrites from APP/PS1, and 5 dendrites from APP/PS1+CP2 mice. For each dendrite, 3-26 spines were analyzed. Each dot represents the average value. *P < 0.05; **P < 0.01; ***P < 0.001; ****P < 0.0001.”

And a related correction has been made in the Methods, under “Chronic CP2 treatment in NTG and symptomatic APP/PS1 mice.”

- The original section read as: “NTG and APP/PS1 female mice (n = 16–21 per group) were given either CP2 (25 mg/kg/day in 0.1% PEG dissolved in drinking water ad lib) or vehicle-containing water (0.1% PEG) starting at 9 months of age as we described in ref. 14. Mice were housed 5 per cage, water consumption and weight were monitored weekly. CP2 concentration was adjusted based on mouse weight/water consumption weekly. Independent groups of mice were continuously treated for 14 months until the age of 23 months. Seven to eight months after CP2 treatment, mice were subjected to the battery of behavior tests, metabolic cages (CLAMS), FDG-PET, ^31^P NMR spectroscopy, and electrophysiology. After mice were sacrificed, tissue and blood were subjected to Western blot analysis, profiling for cytokines/chemokines, next-generation RNA sequencing, immunohistochemistry, electron microscopy examination, and metabolomics as described below.”
- And has now been corrected to: “NTG and APP/PS1 female mice (n = 16–21 per group) were given either CP2 (25 mg/kg/day in 0.1% PEG dissolved in drinking water ad lib) or vehicle-containing water (0.1% PEG) starting at 9 months of age as we described in ref. 14. Mice were housed 5 per cage, water consumption and weight were monitored weekly. CP2 concentration was adjusted based on mouse weight/water consumption weekly. Independent groups of mice were continuously treated for 14 months until the age of 23 months. Seven to eight months after CP2 treatment, mice were subjected to the battery of behavior tests, metabolic cages (CLAMS), ^31^P NMR spectroscopy, and electrophysiology. After mice were sacrificed, tissue and blood were subjected to Western blot analysis, profiling for cytokines/chemokines, next-generation RNA sequencing, immunohistochemistry, electron microscopy examination, and metabolomics as described below.”

There was also an error in the legend to FIg. 1b, which listed the wrong units for CP2 concentration (“mM” rather than “µM”).

- The original legend: “**b** CP2 (light gray bars, 50 mM) does not affect the activity of succinate oxidase (complexes II-III-IV), succinate: cytochrome c reductase (complexes II-III), and ferrocytochrome c oxidase (complex IV only), but significantly inhibits MCI affecting NADH oxidase (complexes I-III-IV) and NADH:ubiquinone (complex I only) in mouse brain mitochondria. Vehicle-dark gray bars.”
- Has now been corrected to state: “**b** CP2 (light gray bars, 50 µM) does not affect the activity of succinate oxidase (complexes II-III-IV), succinate: cytochrome c reductase (complexes II-III), and ferrocytochrome c oxidase (complex IV only), but significantly inhibits MCI affecting NADH oxidase (complexes I-III-IV) and NADH:ubiquinone (complex I only) in mouse brain mitochondria. Vehicle-dark gray bars.”

There was also an error in Fig. 2a, as FDG-PET was conducted in 9 month-old animals after 1–2.6 months of treatment, not after 9 months of treatment as originally indicated.

- The original Figure 2:
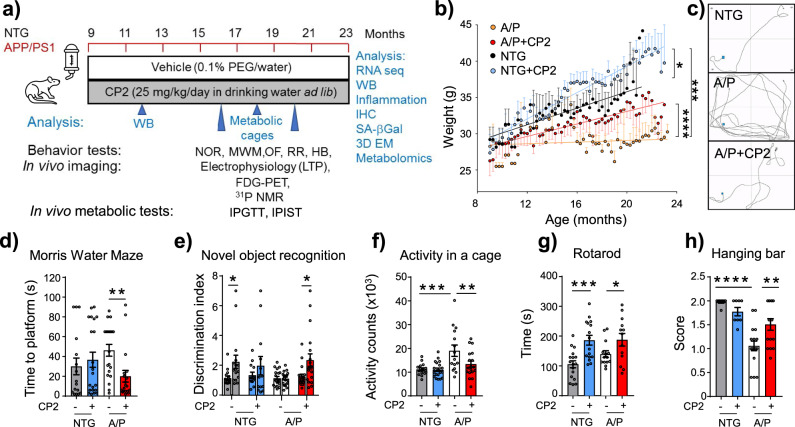
- Has now been corrected:
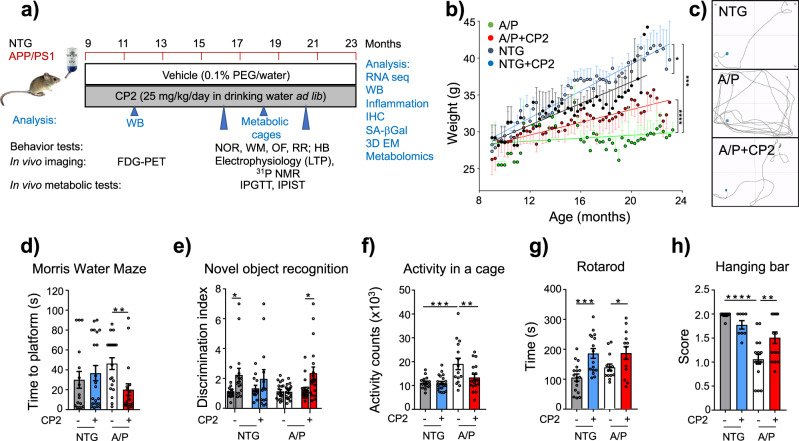

There was also an error in Fig. 3i, which reported the incorrect protein bands for p-AMPK and t-AMPK.

- The original Figure 3:
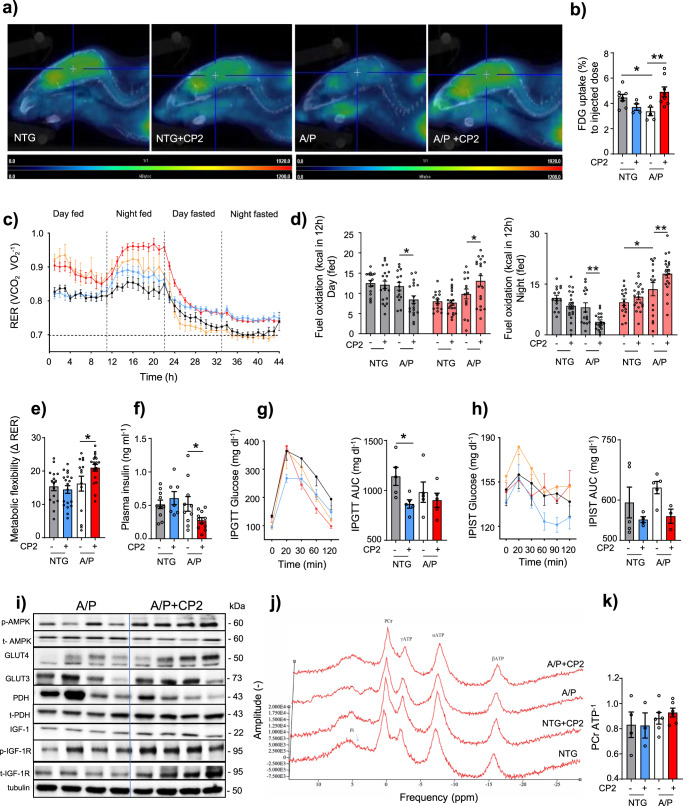
- Has now been corrected:
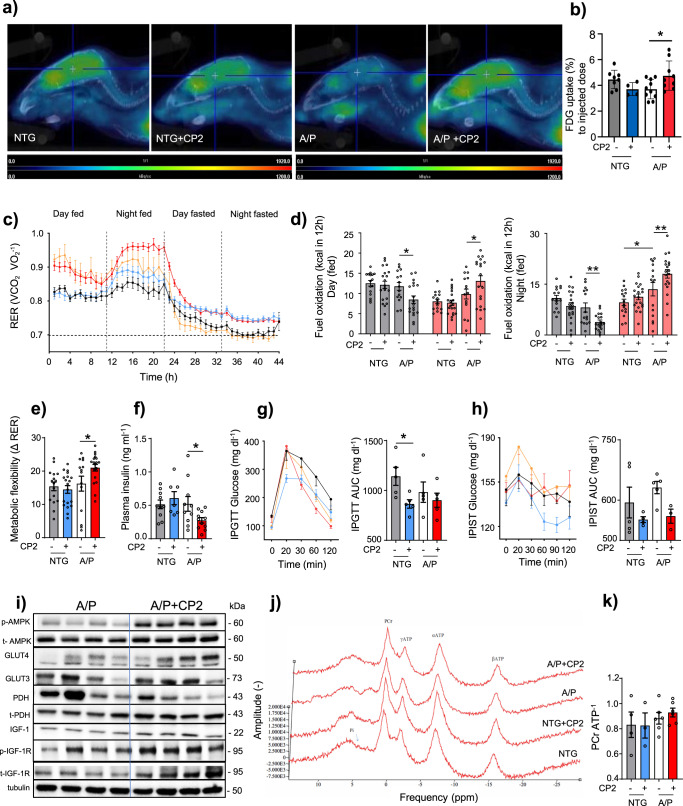

All changes have been made in both the PDF and HTML versions of the article, and its Supplementary Information.

### Supplementary information

Supplementary Information (Corrected)

Supplementary Information (Original)

