# Supplementary material for: Partial inhibition of mitochondrial complex I ameliorates Alzheimer’s disease pathology and cognition in APP/PS1 female mice
Source: Commun Biol. 2021 Jan 8;4:61. doi: 10.1038/s42003-020-01584-y (PMC7794523; doi:10.1038/s42003-020-01584-y)
Supplement: Supplementary file 3 — Description of Additional Supplementary Files [file 42003_2020_1584_MOESM3_ESM.pdf]

Description of Additional Supplementary Files

File name: Supplementary Data 1

Description: Results of kinome profiling for CP2 in the Nanosyn 250 Kinase panel.

File name: Supplementary Data 2

Description: List of DEGs in APP/PS1 *vs.* NTG gene set comparison

File name: Supplementary Data 3

Description: The Gene Ontology enrichment analysis of down-regulated processes in APP/PS1 *vs.* NTG mice.

File name: Supplementary Data 4

Description: The Gene Ontology enrichment analysis of up-regulated processes in APP/PS1 *vs.* NTG mice.

File name: Supplementary Data 5

Description: List of DEGs in APP/PS1 v*s.* APP/PS1+CP2 gene set comparison.

File name: Supplementary Data 6

Descrption: The Gene Ontology enrichment analysis of down-regulated processes in APP/PS1 *vs.* APP/PS1+CP2 comparison.

File name: Supplementary Data 7

Description: The Gene Ontology enrichment analysis of up-regulated processes in APP/PS1 *vs*. APP/PS1+CP2 gene set comparison

File name: Supplementary Data 8

Description: Overlapped DEGs in APP/PS1+CP2 *vs*. APP/PS1 and APP/PS1 *vs*. NTG gene set comparison.

File name: Supplementary Data 9

Description: The Gene Ontology enrichment analysis of functional changes down-regulated by CP2 treatment in APP/PS1 mice to the levels detected in NTG mice.

File name: Supplementary Data 10

Description: The Gene Ontology enrichment analysis of functional changes up-regulated by CP2 treatment in APP/PS1 mice to the levels detected in NTG mice.

File name: Supplementary Data 11

Description: List of down-regulated and up-regulated genes identified in comparison of female AD patients and cognitively normal controls in the AMP-AD data set.

File name: Supplementary Data 12

Description: List of 294 down-regulated DEGs in comparison between NTG *vs.* APP/PS1 mice matched to down-regulated genes identified in the female AMP-AD cohort.

File name: Supplementary Data 13

Description: The Gene Ontology enrichment analysis of down-regulated functional processes in both APP/PS1 mice and females with AD in AMP-AD cohort.

File name: Supplementary Data 14

Description: List of 518 up-regulated DEGs in NTG vs. APP/PS1 mice matching to down-regulated genes identified in the female AMP-AD cohort.

File name: Supplementary Data 15

Description: The Gene Ontology enrichment analysis of up-regulated functional processes in both APP/PS1 mice and female AD patients.

File name: Supplementary Data 16

Description: List of 71 up-regulated DEGs in APP/PS1+CP2 *vs.* APP/PS1 and APP/PS1 *vs.* NTG gene set comparison matched to the up-regulated genes identified in the female AMP-AD cohort.

File name: Supplementary Data 17

Description: The Gene Ontology enrichment analysis of functional processes identified in APP/PS1+CP2 *vs.* APP/PS1 and APP/PS1 *vs.* NTG gene set comparison matched to the up-regulated genes identified in the female AMP-AD cohort.

File name: Supplementary Data 18

Description: List of 57 down-regulated DEGs in APP/PS1+CP2 *vs.* APP/PS1 and APP/PS1 *vs.* NTG gene set comparison, matched to down-regulated genes identified in the female AMP-AD cohort.

File name: Supplementary Data 19

Description: The Gene Ontology enrichment analysis of functional processes identified in APP/PS1+CP2 *vs.* APP/PS1 and APP/PS1 *vs.* NTG gene set comparison matched to the down-r

File name: Supplementary Data 20

Description: Individual P values for data presented in all Figures included in this paper.

File name: Supplementary Data 21

Description: Individual raw values used to generate graphs in the paper.

File name: Supplementary Data 22

Description: R script used for mouse RNA seq data analysis.

File name: Supplementary Data 23

Description: R script used to map mouse to human RNA seq data set.
