# Supplementary material for: Partial inhibition of mitochondrial complex I ameliorates Alzheimer’s disease pathology and cognition in APP/PS1 female mice
Source: Commun Biol. 2021 Jan 8;4:61. doi: 10.1038/s42003-020-01584-y (PMC7794523; doi:10.1038/s42003-020-01584-y)
Supplement: Supplementary file 4 — Supplementary Data 1 [file 42003_2020_1584_MOESM4_ESM.docx]

**Supplementary Data 1.** Results of kinome profiling for CP2 in the Nanosyn 250 Kinase panel. The number represents % inhibition at the concentration tested. Negative numbers represent increased enzyme activity. Numbers below 20 considered to be within the acceptable noise level within the plate.

| Kinase | Conc. Tested (μM) | CP2 |
| --- | --- | --- |
| ABL1 | 1 | -4 |
| ABL1 | 10 | 4 |
| AKT1 | 1 | 3 |
| AKT1 | 10 | 6 |
| AKT2 | 1 | 1 |
| AKT2 | 10 | -4 |
| AKT3 | 1 | 0 |
| AKT3 | 10 | 1 |
| ALK | 1 | 2 |
| ALK | 10 | -2 |
| AMP-A1B1G1 | 1 | -3 |
| AMP-A1B1G1 | 10 | -13 |
| AMP-A2B1G1 | 1 | 3 |
| AMP-A2B1G1 | 10 | 1 |
| ARG | 1 | -1 |
| ARG | 10 | 2 |
| ARK5 | 1 | -3 |
| ARK5 | 10 | 17 |
| AURORA-A | 1 | 3 |
| AURORA-A | 10 | -4 |
| AURORA-B | 1 | 1 |
| AURORA-B | 10 | 7 |
| AURORA-C | 1 | 3 |
| AURORA-C | 10 | -7 |
| AXL | 1 | 2 |
| AXL | 10 | -7 |
| BLK | 1 | 36 |
| BLK | 10 | 34 |
| BMX | 1 | 3 |
| BMX | 10 | -6 |
| BRAF | 1 | 0 |
| BRAF | 10 | -4 |
| BRK | 1 | -3 |
| BRK | 10 | -15 |
| BRSK1 | 1 | -2 |
| BRSK1 | 10 | -4 |
| BRSK2 | 1 | -10 |
| BRSK2 | 10 | -14 |
| BTK | 1 | 1 |
| BTK | 10 | 1 |
| CAMK1A | 1 | 2 |
| CAMK1A | 10 | 1 |
| CAMK1D | 1 | 2 |
| CAMK1D | 10 | -4 |
| CAMK2A | 1 | -1 |
| CAMK2A | 10 | 0 |
| CAMK2B | 1 | 1 |
| CAMK2B | 10 | 4 |
| CAMK2D | 1 | -2 |
| CAMK2D | 10 | -1 |
| CAMK2G | 1 | -5 |
| CAMK2G | 10 | -7 |
| CAMK4 | 1 | 0 |
| CAMK4 | 10 | -6 |
| CDK1 | 1 | -4 |
| CDK1 | 10 | 0 |
| CDK2 | 1 | -3 |
| CDK2 | 10 | -4 |
| CDK2-CYCLINE | 1 | 4 |
| CDK2-CYCLINE | 10 | 1 |
| CDK3-CYCLINE | 1 | 1 |
| CDK3-CYCLINE | 10 | -2 |
| CDK4-CYCLIND | 1 | -1 |
| CDK4-CYCLIND | 10 | 3 |
| CDK5 | 1 | 0 |
| CDK5 | 10 | 3 |
| CDK5-P25 | 1 | 8 |
| CDK5-P25 | 10 | 2 |
| CDK6-CYCLIND3 | 1 | 0 |
| CDK6-CYCLIND3 | 10 | -3 |
| CDK7 | 1 | 4 |
| CDK7 | 10 | 11 |
| CDK9-CYCLINT1 | 1 | 5 |
| CDK9-CYCLINT1 | 10 | -26 |
| CHEK1 | 1 | -1 |
| CHEK1 | 10 | -10 |
| CHEK2 | 1 | 1 |
| CHEK2 | 10 | -1 |
| CK1 | 1 | 1 |
| CK1 | 10 | 19 |
| CK1-EPSILON | 1 | 2 |
| CK1-EPSILON | 10 | 30 |
| CK1-GAMMA1 | 1 | 1 |
| CK1-GAMMA1 | 10 | 19 |
| CK1-GAMMA2 | 1 | -3 |
| CK1-GAMMA2 | 10 | 10 |
| CK1-GAMMA3 | 1 | 1 |
| CK1-GAMMA3 | 10 | 15 |
| CLK1 | 1 | -6 |
| CLK1 | 10 | -6 |
| CLK2 | 1 | -2 |
| CLK2 | 10 | 3 |
| CLK3 | 1 | 1 |
| CLK3 | 10 | -2 |
| CLK4 | 1 | 5 |
| CLK4 | 10 | 24 |
| CRAF | 1 | -1 |
| CRAF | 10 | 0 |
| CSK | 1 | 0 |
| CSK | 10 | -5 |
| DAPK1 | 1 | 0 |
| DAPK1 | 10 | -1 |
| DAPK3 | 1 | -2 |
| DAPK3 | 10 | 0 |
| DCAMKL2 | 1 | -2 |
| DCAMKL2 | 10 | -5 |
| DDR1 | 1 | 3 |
| DDR1 | 10 | 16 |
| DDR2 | 1 | 4 |
| DDR2 | 10 | 2 |
| DYRK1A | 1 | 4 |
| DYRK1A | 10 | 3 |
| DYRK1B | 1 | 2 |
| DYRK1B | 10 | 6 |
| DYRK2 | 1 | -7 |
| DYRK2 | 10 | 1 |
| DYRK3 | 1 | 1 |
| DYRK3 | 10 | 2 |
| DYRK4 | 1 | -2 |
| DYRK4 | 10 | -3 |
| EGFR | 1 | 5 |
| EGFR | 10 | -12 |
| EPH-A1 | 1 | -1 |
| EPH-A1 | 10 | -9 |
| EPH-A2 | 1 | 1 |
| EPH-A2 | 10 | 0 |
| EPH-A3 | 1 | -2 |
| EPH-A3 | 10 | -14 |
| EPH-A4 | 1 | 0 |
| EPH-A4 | 10 | 1 |
| EPH-A5 | 1 | -3 |
| EPH-A5 | 10 | -10 |
| EPH-A8 | 1 | 0 |
| EPH-A8 | 10 | -9 |
| EPH-B1 | 1 | -3 |
| EPH-B1 | 10 | -12 |
| EPH-B2 | 1 | -3 |
| EPH-B2 | 10 | 2 |
| EPH-B3 | 1 | -3 |
| EPH-B3 | 10 | -10 |
| EPH-B4 | 1 | -4 |
| EPH-B4 | 10 | -12 |
| ERB-B2 | 1 | -6 |
| ERB-B2 | 10 | -13 |
| ERB-B4 | 1 | 8 |
| ERB-B4 | 10 | -1 |
| FAK | 1 | 0 |
| FAK | 10 | -3 |
| FER | 1 | -1 |
| FER | 10 | 1 |
| FES | 1 | 5 |
| FES | 10 | -3 |
| FGFR1 | 1 | 5 |
| FGFR1 | 10 | -15 |
| FGFR2 | 1 | 1 |
| FGFR2 | 10 | -3 |
| FGFR4 | 1 | 0 |
| FGFR4 | 10 | -4 |
| FGR | 1 | 0 |
| FGR | 10 | -38 |
| FLT-1 | 1 | 4 |
| FLT-1 | 10 | -23 |
| FLT-3 | 1 | 0 |
| FLT-3 | 10 | 0 |
| FLT-4 | 1 | 1 |
| FLT-4 | 10 | 2 |
| FMS | 1 | 4 |
| FMS | 10 | -1 |
| FRAP1 | 1 | 2 |
| FRAP1 | 10 | 22 |
| FYN | 1 | 1 |
| FYN | 10 | 5 |
| GRK6 | 1 | -2 |
| GRK6 | 10 | -5 |
| GRK7 | 1 | 0 |
| GRK7 | 10 | -12 |
| GSK-3-ALPHA | 1 | 5 |
| GSK-3-ALPHA | 10 | 47 |
| GSK-3-BETA | 1 | -4 |
| GSK-3-BETA | 10 | 21 |
| HASPIN | 1 | 2 |
| HASPIN | 10 | -1 |
| HCK | 1 | 9 |
| HCK | 10 | -1 |
| HIPK1 | 1 | -6 |
| HIPK1 | 10 | 2 |
| HIPK2 | 1 | 0 |
| HIPK2 | 10 | 0 |
| HIPK3 | 1 | -3 |
| HIPK3 | 10 | -1 |
| HIPK4 | 1 | -2 |
| HIPK4 | 10 | -4 |
| IGF1R | 1 | 4 |
| IGF1R | 10 | -4 |
| IKK-ALPHA | 1 | 0 |
| IKK-ALPHA | 10 | -3 |
| IKK-BETA | 1 | 0 |
| IKK-BETA | 10 | 6 |
| IKK-EPSILON | 1 | -1 |
| IKK-EPSILON | 10 | 1 |
| INSR | 1 | -1 |
| INSR | 10 | 1 |
| IRAK1 | 1 | -4 |
| IRAK1 | 10 | -4 |
| IRAK4 | 1 | 0 |
| IRAK4 | 10 | 3 |
| IRR | 1 | 4 |
| IRR | 10 | -1 |
| ITK | 1 | 4 |
| ITK | 10 | -3 |
| JAK1 | 1 | -2 |
| JAK1 | 10 | 2 |
| JAK2 | 1 | 4 |
| JAK2 | 10 | 0 |
| JAK3 | 1 | -2 |
| JAK3 | 10 | -11 |
| JNK1 | 1 | 2 |
| JNK1 | 10 | 6 |
| JNK2 | 1 | 6 |
| JNK2 | 10 | -3 |
| JNK3 | 1 | 4 |
| JNK3 | 10 | 9 |
| KDR | 1 | 6 |
| KDR | 10 | -3 |
| KIT | 1 | 0 |
| KIT | 10 | -6 |
| LATS1 | 1 | 0 |
| LATS1 | 10 | -9 |
| LATS2 | 1 | 0 |
| LATS2 | 10 | 0 |
| LCK | 1 | 2 |
| LCK | 10 | -10 |
| LOK | 1 | 4 |
| LOK | 10 | 14 |
| LRRK2-G2019S | 1 | 0 |
| LRRK2-G2019S | 10 | 3 |
| LTK | 1 | 2 |
| LTK | 10 | -6 |
| LYNA | 1 | 1 |
| LYNA | 10 | -2 |
| LYNB | 1 | 2 |
| LYNB | 10 | -18 |
| MAP4K2 | 1 | 3 |
| MAP4K2 | 10 | 2 |
| MAP4K4 | 1 | 4 |
| MAP4K4 | 10 | 1 |
| MAP4K5 | 1 | 1 |
| MAP4K5 | 10 | 4 |
| MAPK1 | 1 | -1 |
| MAPK1 | 10 | -10 |
| MAPK3 | 1 | -1 |
| MAPK3 | 10 | 3 |
| MAPKAPK-2 | 1 | -4 |
| MAPKAPK-2 | 10 | 2 |
| MAPKAPK-3 | 1 | -1 |
| MAPKAPK-3 | 10 | -3 |
| MARK1 | 1 | 2 |
| MARK1 | 10 | 3 |
| MARK3 | 1 | -4 |
| MARK3 | 10 | -4 |
| MARK4 | 1 | -3 |
| MARK4 | 10 | 0 |
| MEK1 | 1 | 2 |
| MEK1 | 10 | -1 |
| MEK2 | 1 | -1 |
| MEK2 | 10 | 6 |
| MEK3 | 1 | 0 |
| MEK3 | 10 | 2 |
| MELK | 1 | 6 |
| MELK | 10 | 3 |
| MER | 1 | 2 |
| MER | 10 | -1 |
| MET | 1 | 8 |
| MET | 10 | -9 |
| MINK | 1 | 0 |
| MINK | 10 | 10 |
| MKNK1 | 1 | 0 |
| MKNK1 | 10 | 5 |
| MNK2 | 1 | 5 |
| MNK2 | 10 | 5 |
| MRCK-ALPHA | 1 | -2 |
| MRCK-ALPHA | 10 | -3 |
| MRCK-BETA | 1 | 0 |
| MRCK-BETA | 10 | -2 |
| MSK1 | 1 | -1 |
| MSK1 | 10 | -13 |
| MSK2 | 1 | 1 |
| MSK2 | 10 | -11 |
| MSSK1 | 1 | -3 |
| MSSK1 | 10 | 2 |
| MST1 | 1 | 3 |
| MST1 | 10 | 22 |
| MST2 | 1 | 5 |
| MST2 | 10 | 10 |
| MST3 | 1 | -2 |
| MST3 | 10 | -3 |
| MST4 | 1 | -2 |
| MST4 | 10 | -5 |
| MUSK | 1 | 4 |
| MUSK | 10 | -6 |
| NDR2 | 1 | 1 |
| NDR2 | 10 | -4 |
| NDRG1 | 1 | 0 |
| NDRG1 | 10 | -2 |
| NEK1 | 1 | 3 |
| NEK1 | 10 | 7 |
| NEK2 | 1 | 0 |
| NEK2 | 10 | -1 |
| NEK6 | 1 | 4 |
| NEK6 | 10 | 1 |
| NEK7 | 1 | 3 |
| NEK7 | 10 | -20 |
| NEK9 | 1 | 2 |
| NEK9 | 10 | 5 |
| P38-ALPHA | 1 | 4 |
| P38-ALPHA | 10 | 1 |
| P38-BETA | 1 | 3 |
| P38-BETA | 10 | 1 |
| P38-DELTA | 1 | 0 |
| P38-DELTA | 10 | -2 |
| P38-GAMMA | 1 | 0 |
| P38-GAMMA | 10 | -7 |
| P70S6K1 | 1 | 3 |
| P70S6K1 | 10 | -1 |
| P70S6K2 | 1 | 1 |
| P70S6K2 | 10 | -14 |
| PAK1 | 1 | 0 |
| PAK1 | 10 | 2 |
| PAK2 | 1 | 0 |
| PAK2 | 10 | -2 |
| PAK3 | 1 | -1 |
| PAK3 | 10 | -3 |
| PAK4 | 1 | 5 |
| PAK4 | 10 | 10 |
| PAK5 | 1 | 1 |
| PAK5 | 10 | 3 |
| PAK6 | 1 | 4 |
| PAK6 | 10 | -3 |
| PAR-1B-ALPHA | 1 | -3 |
| PAR-1B-ALPHA | 10 | 7 |
| PASK | 1 | -5 |
| PASK | 10 | 2 |
| PDGFR-ALPHA | 1 | 3 |
| PDGFR-ALPHA | 10 | 3 |
| PDGFR-BETA | 1 | 4 |
| PDGFR-BETA | 10 | -1 |
| PDK1 | 1 | -4 |
| PDK1 | 10 | 0 |
| PERK | 1 | 2 |
| PERK | 10 | -14 |
| PHK-GAMMA1 | 1 | 3 |
| PHK-GAMMA1 | 10 | 0 |
| PHK-GAMMA2 | 1 | 1 |
| PHK-GAMMA2 | 10 | 5 |
| PI3-KINASE-ALPHA | 1 | 2 |
| PI3-KINASE-ALPHA | 10 | 7 |
| PI4-K-BETA | 1 | 0 |
| PI4-K-BETA | 10 | -3 |
| PIM-1-KINASE | 1 | 0 |
| PIM-1-KINASE | 10 | 1 |
| PIM2 | 1 | 1 |
| PIM2 | 10 | -2 |
| PIM3 | 1 | -1 |
| PIM3 | 10 | 0 |
| PKA | 1 | 2 |
| PKA | 10 | -11 |
| PKACB | 1 | -1 |
| PKACB | 10 | 0 |
| PKC-ALPHA | 1 | 1 |
| PKC-ALPHA | 10 | 0 |
| PKC-BETA1 | 1 | 0 |
| PKC-BETA1 | 10 | 8 |
| PKC-BETA2 | 1 | 2 |
| PKC-BETA2 | 10 | 0 |
| PKC-ETA | 1 | -2 |
| PKC-ETA | 10 | 1 |
| PKC-GAMMA | 1 | 2 |
| PKC-GAMMA | 10 | 1 |
| PKC-IOTA | 1 | -6 |
| PKC-IOTA | 10 | 3 |
| PKC-THETA | 1 | -3 |
| PKC-THETA | 10 | -1 |
| PKC-ZETA | 1 | -1 |
| PKC-ZETA | 10 | -3 |
| PKN1 | 1 | -1 |
| PKN1 | 10 | -4 |
| PKN2 | 1 | 2 |
| PKN2 | 10 | 1 |
| PLK1 | 1 | -1 |
| PLK1 | 10 | -19 |
| PLK3 | 1 | -5 |
| PLK3 | 10 | 14 |
| PLK4 | 1 | -9 |
| PLK4 | 10 | -11 |
| PRAK | 1 | -3 |
| PRAK | 10 | -8 |
| PRKACA | 1 | -2 |
| PRKACA | 10 | -2 |
| PRKD1 | 1 | 7 |
| PRKD1 | 10 | -4 |
| PRKD2 | 1 | 0 |
| PRKD2 | 10 | 2 |
| PRKD3 | 1 | 5 |
| PRKD3 | 10 | -3 |
| PRKG1 | 1 | 2 |
| PRKG1 | 10 | 4 |
| PRKX | 1 | 0 |
| PRKX | 10 | -1 |
| PTK5 | 1 | -1 |
| PTK5 | 10 | -3 |
| PYK2 | 1 | 5 |
| PYK2 | 10 | -8 |
| RET | 1 | 3 |
| RET | 10 | -4 |
| RIPK2 | 1 | 0 |
| RIPK2 | 10 | -5 |
| ROCK1 | 1 | 5 |
| ROCK1 | 10 | -1 |
| ROCK2 | 1 | 7 |
| ROCK2 | 10 | 16 |
| RON | 1 | 8 |
| RON | 10 | -12 |
| ROS | 1 | 4 |
| ROS | 10 | -5 |
| RSK1 | 1 | 2 |
| RSK1 | 10 | 0 |
| RSK2 | 1 | -2 |
| RSK2 | 10 | -6 |
| RSK3 | 1 | -3 |
| RSK3 | 10 | -1 |
| RSK4 | 1 | -1 |
| RSK4 | 10 | -1 |
| SGK1 | 1 | 1 |
| SGK1 | 10 | 1 |
| SGK2 | 1 | -4 |
| SGK2 | 10 | 1 |
| SGK3 | 1 | 5 |
| SGK3 | 10 | -5 |
| SIK | 1 | 4 |
| SIK | 10 | 1 |
| SLK | 1 | -2 |
| SLK | 10 | 4 |
| SNF1LK2 | 1 | 4 |
| SNF1LK2 | 10 | 1 |
| SPHK1 | 1 | 3 |
| SPHK1 | 10 | 0 |
| SPHK2 | 1 | 4 |
| SPHK2 | 10 | -4 |
| SRC | 1 | 2 |
| SRC | 10 | -18 |
| SRMS | 1 | -6 |
| SRMS | 10 | -10 |
| SRPK1 | 1 | 3 |
| SRPK1 | 10 | -8 |
| SRPK2 | 1 | -3 |
| SRPK2 | 10 | 0 |
| STK16 | 1 | 1 |
| STK16 | 10 | -5 |
| STK25 | 1 | -1 |
| STK25 | 10 | 2 |
| SYK | 1 | 6 |
| SYK | 10 | -15 |
| TAK1-TAB1 | 1 | -1 |
| TAK1-TAB1 | 10 | -1 |
| TAOK2 | 1 | -2 |
| TAOK2 | 10 | -1 |
| TAOK3 | 1 | 3 |
| TAOK3 | 10 | -2 |
| TBK1 | 1 | -2 |
| TBK1 | 10 | -7 |
| TEC | 1 | 2 |
| TEC | 10 | 1 |
| TIE2 | 1 | 0 |
| TIE2 | 10 | -9 |
| TNIK | 1 | 1 |
| TNIK | 10 | 1 |
| TNK2 | 1 | 3 |
| TNK2 | 10 | 2 |
| TRKA | 1 | 2 |
| TRKA | 10 | -5 |
| TRKB | 1 | 5 |
| TRKB | 10 | -3 |
| TRKC | 1 | 3 |
| TRKC | 10 | -3 |
| TSSK1 | 1 | -2 |
| TSSK1 | 10 | 7 |
| TSSK2 | 1 | -2 |
| TSSK2 | 10 | 1 |
| TTK | 1 | 2 |
| TTK | 10 | 4 |
| TXK | 1 | 6 |
| TXK | 10 | -5 |
| TYK2 | 1 | 4 |
| TYK2 | 10 | -7 |
| TYRO3 | 1 | 0 |
| TYRO3 | 10 | -4 |
| YES | 1 | -1 |
| YES | 10 | -4 |
| ZAP70 | 1 | -15 |
| ZAP70 | 10 | -3 |
