# Supplementary material for: Partial inhibition of mitochondrial complex I ameliorates Alzheimer’s disease pathology and cognition in APP/PS1 female mice
Source: Commun Biol. 2021 Jan 8;4:61. doi: 10.1038/s42003-020-01584-y (PMC7794523; doi:10.1038/s42003-020-01584-y)
Supplement: Supplementary file 25 — Supplementary Data 22 [file 42003_2020_1584_MOESM25_ESM.pdf]

Supplementary Data 22. R script used for mouse RNA seq data analysis

```
args = commandArgs(TRUE)
input = args[1]
group = args[2]
baseDir = args[3]

if (length(args) < 3){
  writeLines ("Please check arguments.\nUsage:\nRscript
 deseq.R input group baseDir\nFor group file, create a
 tab separated file having headers, sample names and the
 group they belong to. For example, if there are 2
 samples named sampleA and sampleB:\n
             samples  group\n
             sampleA  g1\n
             sampleB  g2\n");
  quit()
}

library(DESeq2)
library(rlocal)
library(ggplot2)
library(genefilter)
library(gplots)
library(ggplot2)
library(ggrepel)
library(pheatmap)
library(plyr)
library(dplyr)
library(RColorBrewer)
library(edgeR)

##IMP##
#Create a graphs directory
out=gsub(".filtered","",tools::file_path_sans_ext(basena
 me(input)))

config_path=paste(baseDir,"/config/",sep="")
```

```
results_path=paste(baseDir,"/results/",sep="")
graphs_path=paste(baseDir,"/graphs/",out,"/",sep="")
setwd(config_path)



data<-read.table(file=input, header=TRUE,
 stringsAsFactors=TRUE, row.names=1, sep="\t")

coldata<-read.table(file=group,header=TRUE,
 stringsAsFactors=TRUE, row.names=1,
 colClasses=c("factor","factor"), sep="\t")

count=NULL
for(sample in rownames(coldata)){
       i=1;
       for(ref_cols in colnames(data)){
            if(sample == ref_cols){
                  index=i
                  #print(index)
                  count=cbind(count,data[,index])
                  break
            }
            i=i+1
       }
}

colnames(count)=rownames(coldata)
rownames(count)=rownames(data)
sample_names<-colnames(count)


#May be needed
#colnames(count) <- NULL

dds_pre <- DESeqDataSetFromMatrix(countData = count,
```

```
 colData=coldata, design= ~group)
dds_pre$group <- relevel(dds_pre$group, "NTG.Tr.Adult")

#CPM>1 calc
main=NULL
for(i in unique(coldata[,1])){
      a=rownames(subset(coldata, group==i))
      keep=rowSums(fpm(dds_pre[,a], robust=TRUE) > 1) ==
 length(a)
      main=cbind(main,keep)
}

dds=dds_pre[rowSums(main[,1:2]=="TRUE") >= 1,]

dds <- DESeq(dds)

##For multi-sample analysis
res <- results(dds, pAdjustMethod="BH")
##summary(res,0.05)
res.05 <- results(dds, alpha=0.05, lfcThreshold=1.5,
 pAdjustMethod="BH")
print("At +/- 1.5 log2fc threshold and padj<0.05:")
summary(res.05)

##Order according to adj pval
#resOrdered <- res[order(res$padj),]
mcols(res)$description

##Hits with adj Pval 0.05 (alpha=FDR=0.1 by default,
 change to 0.05 if padj cutoff should be 0.05)
#res05 <- results(dds, alpha=0.05)
#summary(res05)

data$GeneID <- rownames(data)
res$GeneID <- rownames(res)
res.df=as.data.frame(res)
```

```
## Write results
outfile=paste(out,".tsv",sep="")
full=merge(data,res.df,by.x="GeneID",by.y="GeneID")
write.table(as.data.frame(full),
 file=paste(results_path,outfile,sep=""), sep="\t",
 row.names=FALSE, quote=F)

##Transformed counts
#vsd <- vst(dds, blind=FALSE) ##Turn off if unpaired
 samples/no replicates
rld <- rlog( dds )
ntd <- normTransform(dds)

## Histogram of pval
h=hist(res$pvalue, breaks=20)
maxh <- max(h$counts)
strh <- strheight('W')
strw <- strwidth(max(h$counts))
png(file=paste(graphs_path,"pval.hist.png",sep=""),width
 =1000,height=1000)
hist(res$pvalue, breaks=20, col="grey", ylim=c(0, maxh +
 strh + strw), xlab="P-value", ylab="Counts",axes=TRUE,
 plot=TRUE, main="Histogram of P-values")
text(h$mids, strh + h$counts, labels=h$counts, adj=c(0,
 0.5), srt=90)
dev.off()

## Hisotgram of adjp
h=hist(res$padj, breaks=20)
maxh <- max(h$counts)
strh <- strheight('W')
strw <- strwidth(max(h$counts))
png(file=paste(graphs_path,"padj.hist.png",sep=""),width
 =1000,height=1000)
hist(res$padj,breaks=20, col="grey", ylim=c(0, maxh +
```

```
 strh + strw), xlab="Adjusted p-value",
 ylab="Counts",axes=TRUE, plot=TRUE, main="Histogram of
 adjusted p-values")
text(h$mids, strh + h$counts, labels=h$counts, adj=c(0,
 0.5), srt=90)
dev.off()

#MA Plot shrink logFC values
rN=resultsNames(dds)
resLFC <- lfcShrink(dds, coef=rN[2])
png(file=paste(graphs_path,"ma.plot.png",sep=""),width=1
 000,height=1000)
plotMA(resLFC, alpha=0.05, ylim=c(-10,10))
dev.off()

#Boxplot-raw
png(file=paste(graphs_path,"boxplot.raw.png",sep=""),wid
 th=1000,height=1000)
boxplot(log(1+count),las=2)
dev.off()

#Boxplot-normalized
png(file=paste(graphs_path,"boxplot.norm.png",sep=""),wi
 dth=1000,height=1000)
boxplot(log(1+assay(rld)),las=2)
dev.off()


### Heatmap of count matrix ###
df <- as.data.frame(colData(dds)["group"])
rownames(df)=colnames(dds)
pheatmap_holder=pheatmap(as.matrix(fpm(dds,robust=TRUE))
 , scale="row", show_rownames=FALSE, annotation_col=df,
 color = rev(colorRampPalette(c("red", "black", "green"))
 (n = 299)), clustering_distance_cols="correlation",
 clustering_distance_rows="correlation",
```

```
 clustering_method="ward.D")

save_pheatmap_png <- function(x, filename, width=1200,
 height=1200) {
  png(filename, width = width, height = height)
  grid::grid.newpage()
  grid::grid.draw(x$gtable)
  dev.off()
 }

save_pheatmap_png(pheatmap_holder,
 paste(graphs_path,"heatmap.png",sep=""))

#mat=log(1+count)
#high.cv=tail(sort(apply(mat,1, function(x) sd(x)/
 mean(x))),5000)
#gene.names=names(high.cv)
#sub.mat=as.matrix(mat[gene.names,])
#heatmap(sub.mat, scale="row", show_rownames=FALSE,
 annotation_col=df, color = rev(colorRampPalette(c("red",
 "black", "green"))(n = 299)),
 clustering_distance_cols="correlation",
 clustering_distance_rows="correlation", clustering>
 ethod="ward.D")

####Heatmap - Top 50####
#rpkm=16:23
#rpkm <- full[ , grepl( "_rpkm" , names( full ) ) ]
rpkm <- which(grepl( ".RPKM" , names( full ) ))

data_top=head(arrange(full, padj, desc(log2FoldChange)),
 50)
#data_top=head(order(full$pvalue,
 abs(full$log2FoldChange), decreasing=TRUE),50)
#genes=make.unique(as.character(full[data_top,6]))
genes=make.unique(as.character(data_top[,3]))
```

```
#data_plot=full[data_top,rpkm]
data_plot=data_top[,rpkm]
rownames(data_plot)=genes
colnames(data_plot)=gsub(".RPKM","",colnames(data_plot))

png(file=paste(graphs_path,"top.50.png",sep=""),
 width=1200, height=1200)
heatmap.2(as.matrix(data_plot),
 ColSideColors=c(rep("aquamarine",sum(coldata[,
 1]==unique(coldata[,1])
 [1])),rep("darkgoldenrod1",sum(coldata[,
 1]==unique(coldata[,1])[2]))), Colv=FALSE, Rowv=FALSE,
 scale="row",trace="none",
 dendrogram="none",margins=c(5,8),cexCol=1,cexRow=1, col
 =rev(colorRampPalette(c("red", "black", "green"))(n =
 299)))
legend("topright",legend = unique(coldata$group),col
 =c("aquamarine","darkgoldenrod1") , lty= 5, lwd =
 10,cex=1)
dev.off()

##2D PCA
colnames(count)=sample_names
grp <- as.vector(coldata$group)
sample.labels=unique(grp)
a=length(unique(grp))
color.scheme <-
 colorRampPalette( c( "indianred2","seashell3",
 "blue" ) )(a)
grp_color=mapvalues(as.vector(coldata$group),from=sample
 .labels,to=color.scheme)


png(file=paste(graphs_path,"2D.PCA.png",sep=""),width=10
 00, height=1000)
```

```
plotMDS(full[,rpkm],
 pch=20,labels=gsub(".RPKM","",colnames(full[,rpkm])) ,ma
 in="MDS Plot", ndim=2, dim.plot=c(1,2), cex=1,
 cex.lab=1.5, cex.axis=1.5, cex.main=1.5, cex.sub=1.5,
 col=grp_color, xlab="Component 1", ylab="Component 2")
#toplot <- data.frame(Dim1 = mds$x, Dim2 = mds$y, Groups
 =grp)
#gg=ggplot(toplot, aes(Dim1, Dim2, colour = Groups)) +
 geom_point(size=4)
#gg + geom_point(shape=21,color="black",size=4,
 stroke=1) + xlab("Component 1") + ylab("Component 2")
#ggsave(paste(graphs_path,"2D.PCA.png",sep=""),units="in
 ", width=10,height=10, dpi=800)

legend("topleft", legend = sample.labels, col =
 color.scheme, lty=1,lwd=10)
dev.off()

##Volcano plot
dataset<-mutate(full, sig=ifelse((full$padj<=0.05 &
 abs(full$log2FoldChange)>=1), "padj<=0.05 AND
 abs(logFC)>=1", ifelse((full$pvalue<=0.05 &
 abs(full$log2FoldChange)>=1), "pval<=0.05 AND
 abs(logFC)>=1", "Not.Sig")))

input<-filter(dataset, padj<=0.05 &
 abs(log2FoldChange)>=1)
input.sorted<-input[order(abs(input$log2FoldChange)),]

png(file=paste(graphs_path,"volcano.png",sep=""),width=1
 000, height=1000)
ggplot(dataset, aes(log2FoldChange, -log10(pvalue))) +
 geom_point(aes(col=sig)) +
 scale_color_manual(values=c("black","red","green")) +
 geom_text_repel(data=tail(input.sorted,50),
 aes(label=make.unique(as.character(GeneName)))
```

```
#ggsave(paste(graphs_path,"volcano.png",sep=""))
dev.off()

##as.matrix(data[head(order(RowVar(data[31:55]),decreasi
 ng = TRUE),5000),31:55])


#vars=rowVars(data[,31:55])
#vars_order=order(vars,decreasing=TRUE)
#select=head(data[vars_order,31:55],5000)
#colnames(select)=gsub(".RPKM","",colnames(select))
#png("top.5000.genes.png", width=1200, height=1200)
#heatmap.2(as.matrix(select),
 ColSideColors=c(rep("aquamarine",sum(coldata[,
 1]==unique(coldata[,1])
 [1])),rep("darkgoldenrod1",sum(coldata[,
 1]==unique(coldata[,1])[2])),rep("khaki1",sum(coldata[,
 1]==unique(coldata[,1])
 [2])),rep("lightpink1",sum(coldata[,1]==unique(coldata[,
 1])[2])),rep("mediumorchid",sum(coldata[,
 1]==unique(coldata[,1])[2]))), Colv=TRUE, Rowv=FALSE,
 labRow = FALSE, scale="row",trace="none",
 dendrogram="both",margins=c(5,8),cexCol=1,cexRow=1, col
 =rev(colorRampPalette(c("red", "black", "green"))(n =
 299)))
#legend("topright",legend = unique(coldata$group),col
 =c("aquamarine","darkgoldenrod1","khaki1","lightpink1","
 mediumorchid") , lty= 5, lwd = 10,cex=1)
#dev.off()
```
