# Supplementary material for: Partial inhibition of mitochondrial complex I ameliorates Alzheimer’s disease pathology and cognition in APP/PS1 female mice
Source: Commun Biol. 2021 Jan 8;4:61. doi: 10.1038/s42003-020-01584-y (PMC7794523; doi:10.1038/s42003-020-01584-y)
Supplement: Supplementary file 26 — Supplementary Data 23 [file 42003_2020_1584_MOESM26_ESM.pdf]

Supplementary Data 23. R script used to map mouse to human RNA seq data set

```
# read in the human and mouse data
human.data <- read.table('humman.genes.txt')
mouse.data <- read.table('mouse.genes.txt')


# merge the human data and mouse data by mapping mouse gene symbols to
human gene symbols
mouse.data$mouse.geneID <- toupper(mouse.data$mouse.geneID)

mapped.data <- merge(human.data, mouse.data, by.x='human.geneID',
by.y='mouse.geneID')

write.table(mapped.data, file='merged.data.txt')
```
