# Supplementary material for: Author Correction: Partial inhibition of mitochondrial complex I ameliorates Alzheimer’s disease pathology and cognition in APP/PS1 female mice
Source: Commun Biol. 2024 Feb 26;7:234. doi: 10.1038/s42003-024-05810-9 (PMC10897414; doi:10.1038/s42003-024-05810-9)
Supplement: Supplementary file 2 — Supplementary Information (Original) [file 42003_2024_5810_MOESM2_ESM.pdf]

Supplementary Information

Partial inhibition of mitochondrial complex I ameliorates Alzheimer's disease pathology and cognition in APP/PS1 female mice

Andrea Stojakovic[1,*], Sergey Trushin[1,*], Anthony Sheu[2,*], Layla Khalili[1], Su-Youne Chang[3,4], Xing Li[5], Trace Christensen[6], Jeffrey L. Salisbury[6,7], Rachel E. Geroux[1], Benjamin Gateno[1], Padraig J. Flannery[1], Mrunal Dehankar[5], Cory C. Funk[8], Jordan Wilkins[1], Anna Stepanova[9], Tara O'Hagan[9], Alexander Galkin[9], Jarred Nesbitt[1], Xiujuan Zhu[1], Utkarsh Tripathi[1], Slobodan Macura[7], Tamar Tchkonia[10], Tamar Pirtskhalava[10], James L. Kirkland[10], Rachel A. Kudgus[11], Renee A. Schoon[11], Joel M. Reid[11], Yu Yamazaki[12], Takahisa Kanekiyo[12], Song Zhang[13], Emirhan Nemutlu[14], Petras Dzeja[13], Adam Jaspersen[6], Christopher Ye In Kwon[2], Michael K. Lee[2,#], Eugenia Trushina[1,11,#]

[1]Department of Neurology, Mayo Clinic, 200 First St. SW, Rochester, MN 55905, USA

[2]Institute for Translational Neuroscience, University of Minnesota Twin Cities, 2101 6th Street SE, Minneapolis, MN 55455, USA

[3]Department of Neurologic Surgery, Mayo Clinic, 200 First St. SW, Rochester, MN 55905, USA

[4]Department of Physiology and Biomedical Engineering, Mayo Clinic, 200 First St. SW, Rochester, MN 55905, USA

[5]Division of Biomedical Statistics and Informatics, Department of Health Sciences Research, Mayo Clinic, 200 First St. SW, Rochester, MN 55905, USA

[6]Microscopy and Cell Analysis Core, Mayo Clinic, 200 First St. SW, Rochester, MN 55905, USA

[7]Department of Biochemistry and Molecular Biology, Mayo Clinic, 200 First St. SW, Rochester, MN 55905, USA

[8]Institute for Systems Biology, Seattle, WA, 98109-5263, USA

[9]Division of Neonatology, Department of Pediatrics, Columbia University, 116th St & Broadway New York, NY 10027, USA

[10]Robert and Arlene Kogod Center on Aging, Mayo Clinic, 200 First St. SW, Rochester, MN 55905, USA

[11]Department of Molecular Pharmacology and Experimental Therapeutics, Mayo Clinic, 200 First St. SW, Rochester, MN 55905, USA

[12]Department of Neuroscience, Mayo Clinic, 4500 San Pablo Road, Jacksonville, FL 32224, USA

[13]Department of Cardiovascular Medicine, Mayo Clinic, 200 First St. SW, Rochester, MN 55905, USA

[14]Faculty of Pharmacy, Department of Analytical Chemistry, Hacettepe University, Sihhiye, Ankara, 06100, Turkey

*These authors contributed equally

#These authors jointly supervised this work

Corresponding author: Eugenia Trushina, Ph.D.

200 First Street SW, Guggenheim Bld

Room 1542B

Rochester, MN 55905

USA

Phone: 507-284-8197

trushina.eugenia@mayo.edu

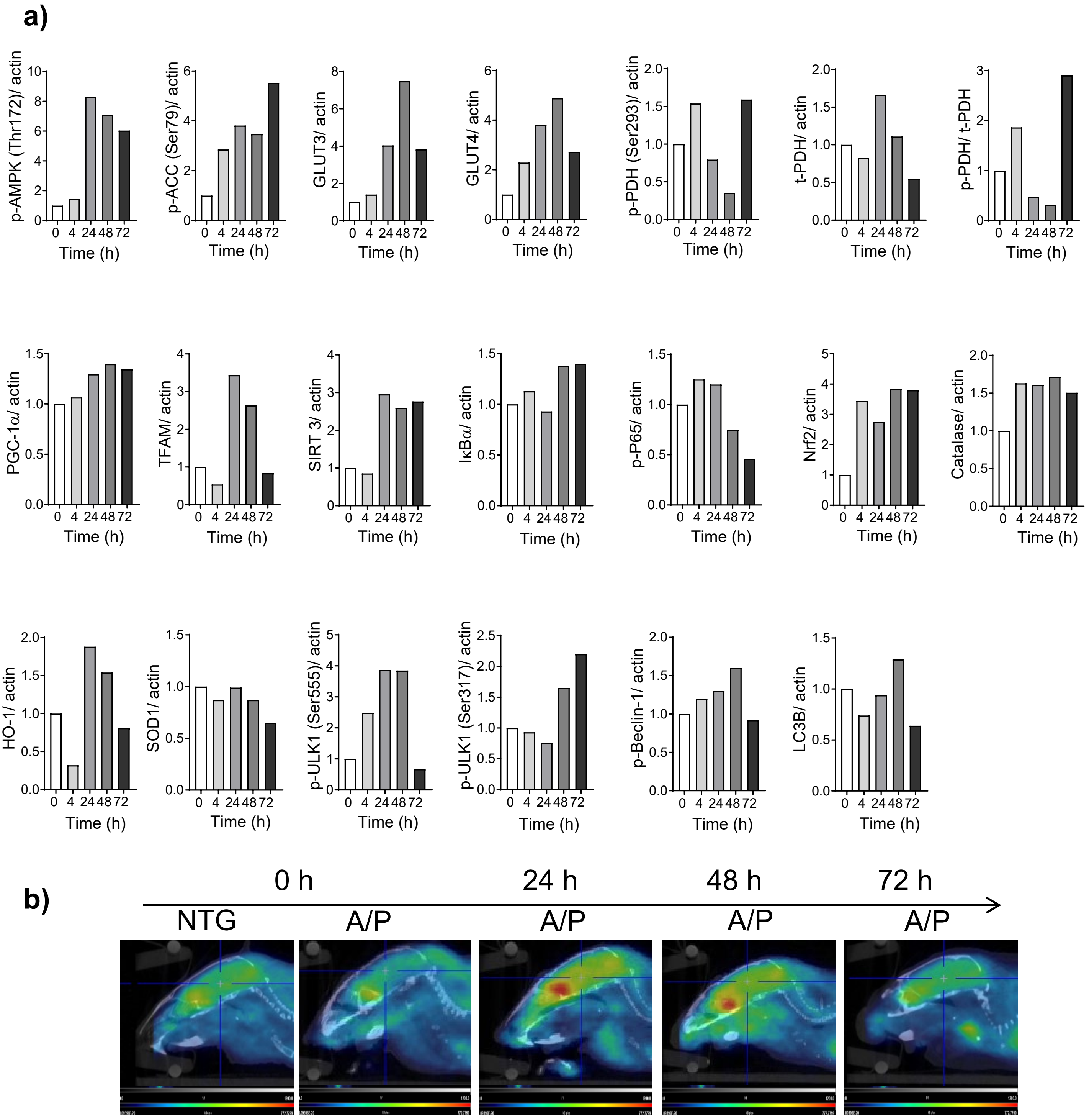

Supplementary Figure 1. CP2 activates AMPK-dependent neuroprotective pathways in symptomatic APP/PS1 mice and improves glucose uptake in the brain. a) Quantification of time-dependent changes in levels of key proteins involved in multiple neuroprotective pathways in the brain of symptomatic APP/PS1 mice acutely gavaged with CP2 from experiments described in Figure 1**e** (25 mg/kg, *n* = 1 *per* time point) using Western blot analysis. **b)** Representative FDG-PET images collected from the independent cohort of APP/PS1 mice (9-10-month-old) before CP2 administration and 24, 48 and 72 h after a single dose via gavage (25 mg/kg, Figure 1**e**,**h**). NTG, untreated non-transgenic age- and sex-matched littermates. NTG, *n* = 3 female mice *per* group and APP/PS1, *n* = 3 female mice *per* group.

Western blots for Fig. 1

Figure 1f

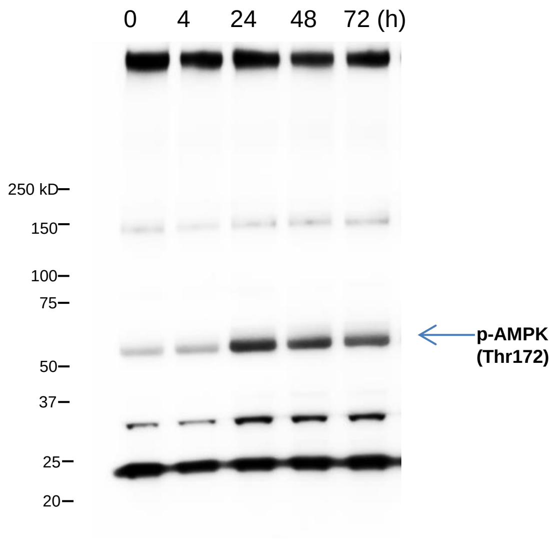

Figure 1f

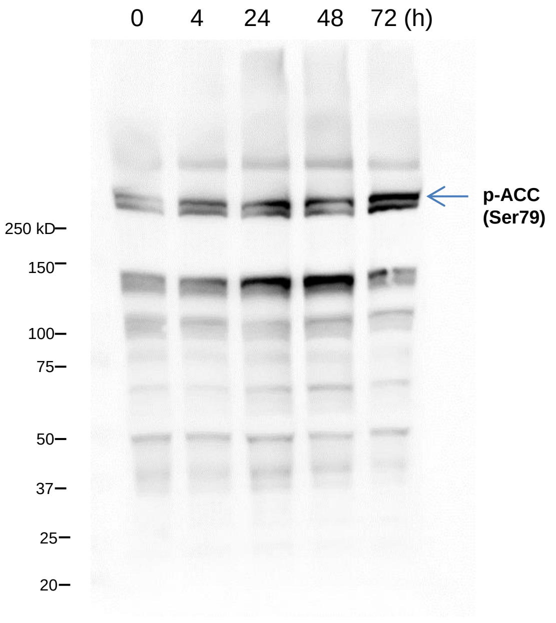

Figure 1g

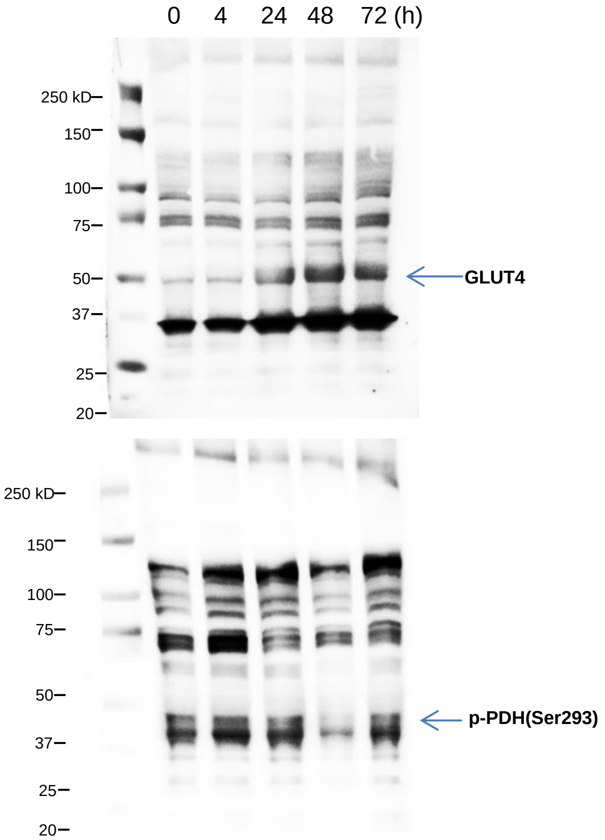

Figure 1g

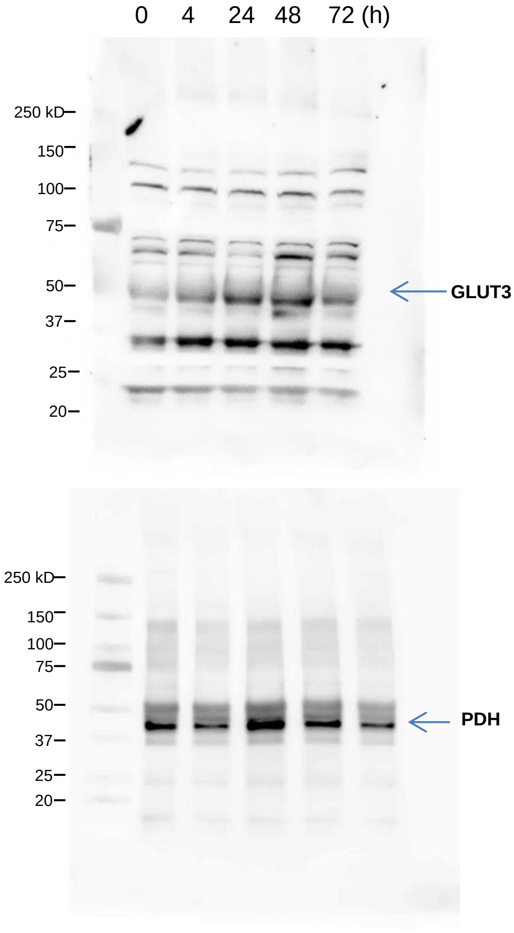

Western blots for Fig. 1

Figure 1i

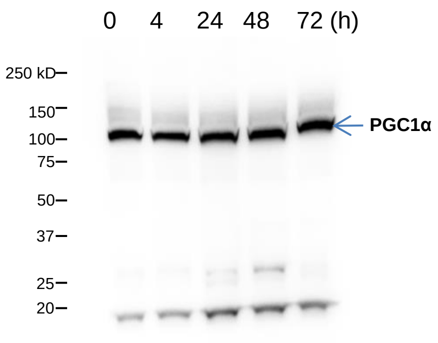

Figure 1i

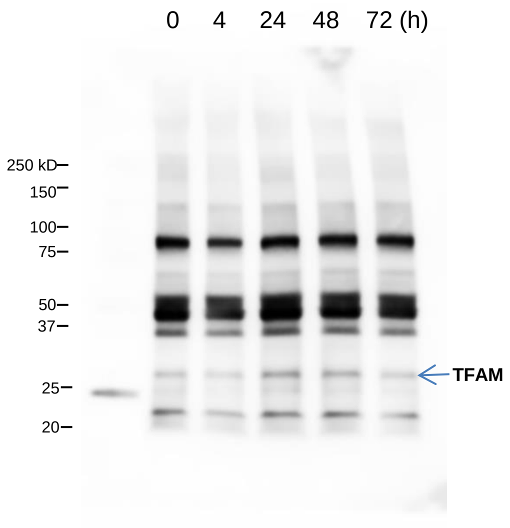

Figure 1i

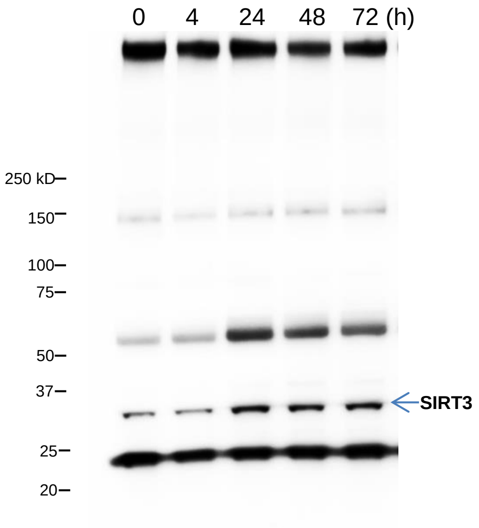

Figure 1j

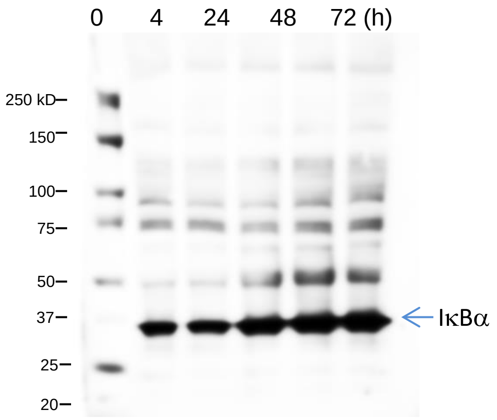

Figure 1j

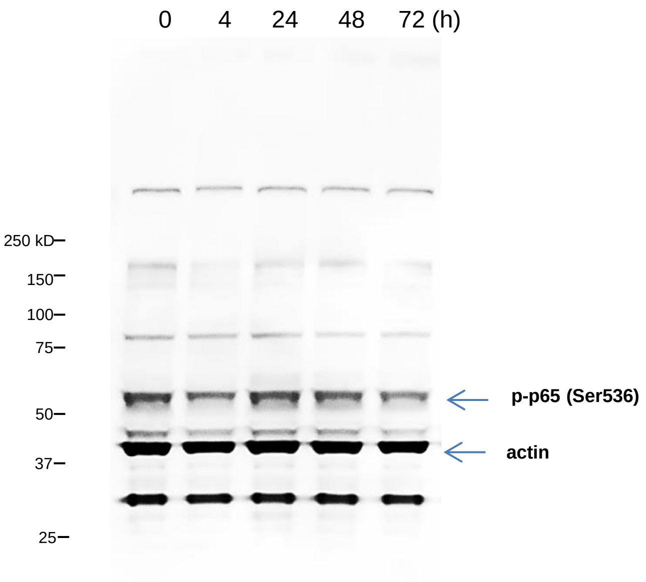

Figure 1k

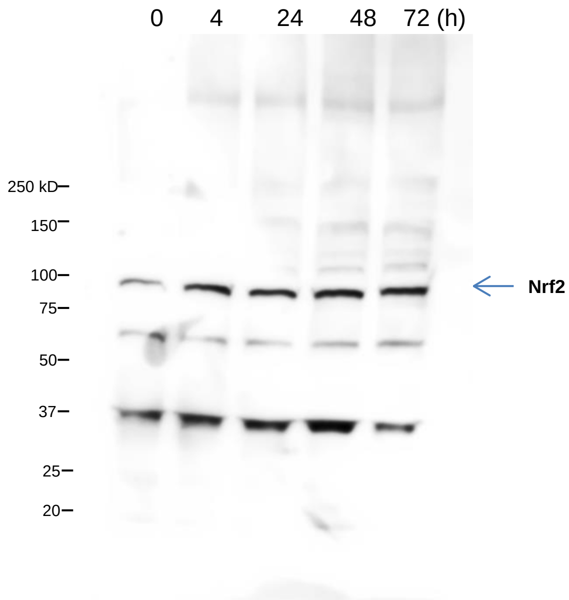

Figure 1k

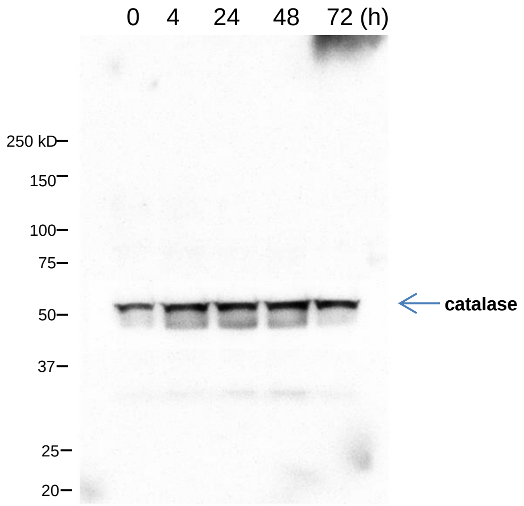

Western blots for Fig. 1

Figure 1k

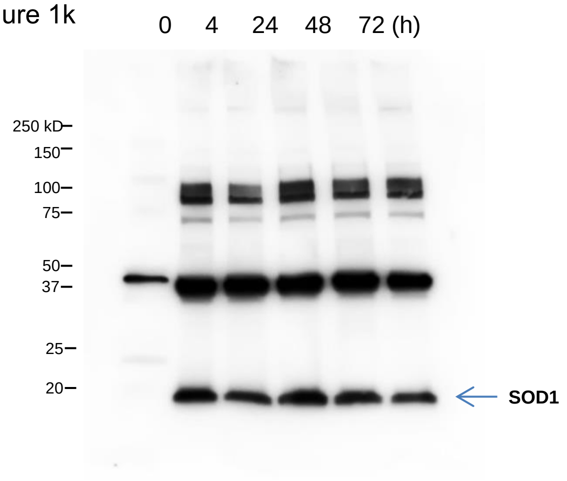

Figure 1k

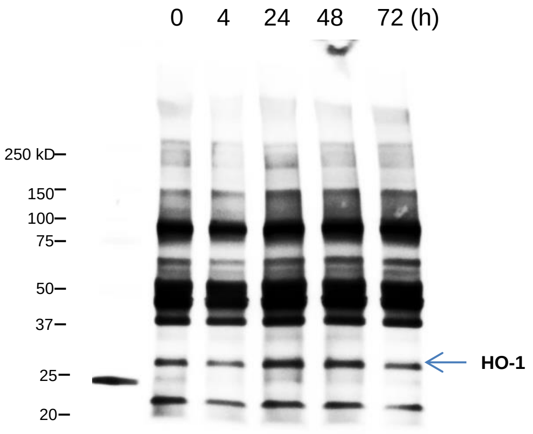

Figure 1l

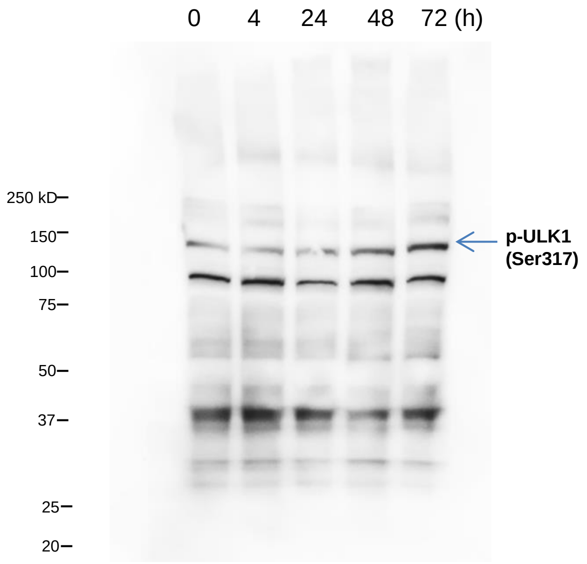

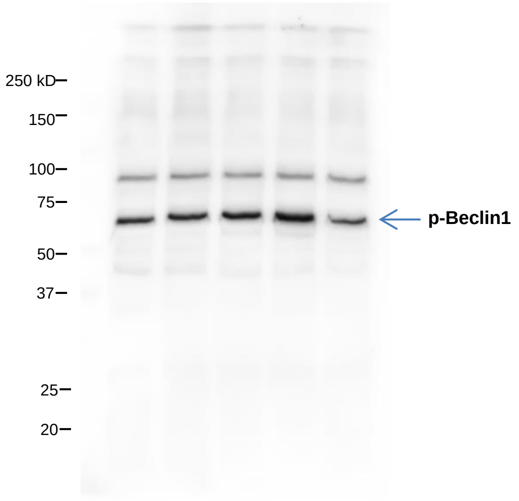

Figure 1l

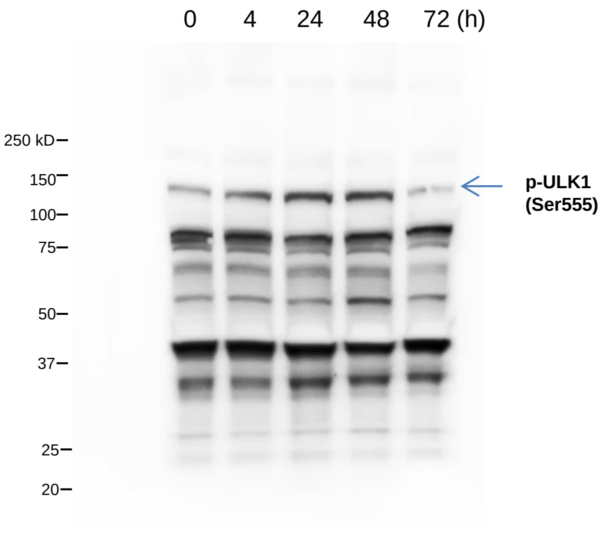

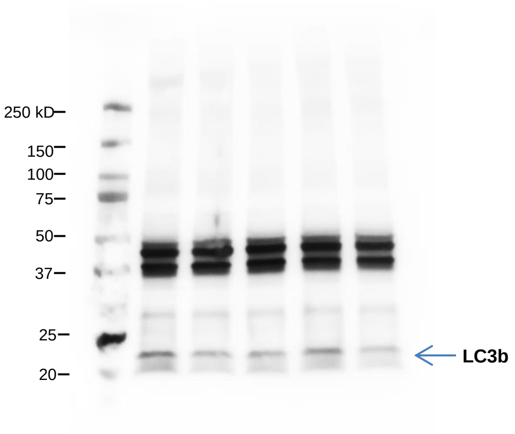

Supplementary Figure 2. Original uncropped Western blot images for Figure 1e-l. Time course of the expression of key proteins in each of the AMPK-dependent neuroprotective pathways depicted in Figure 1**d** in the brain tissue of symptomatic APP/PS1 mice 9 months of age acutely gavaged with 25 mg/kg of CP2. One mouse was taken for each time point. Blue arrows indicate the bands shown in Figure 1**f-l**.

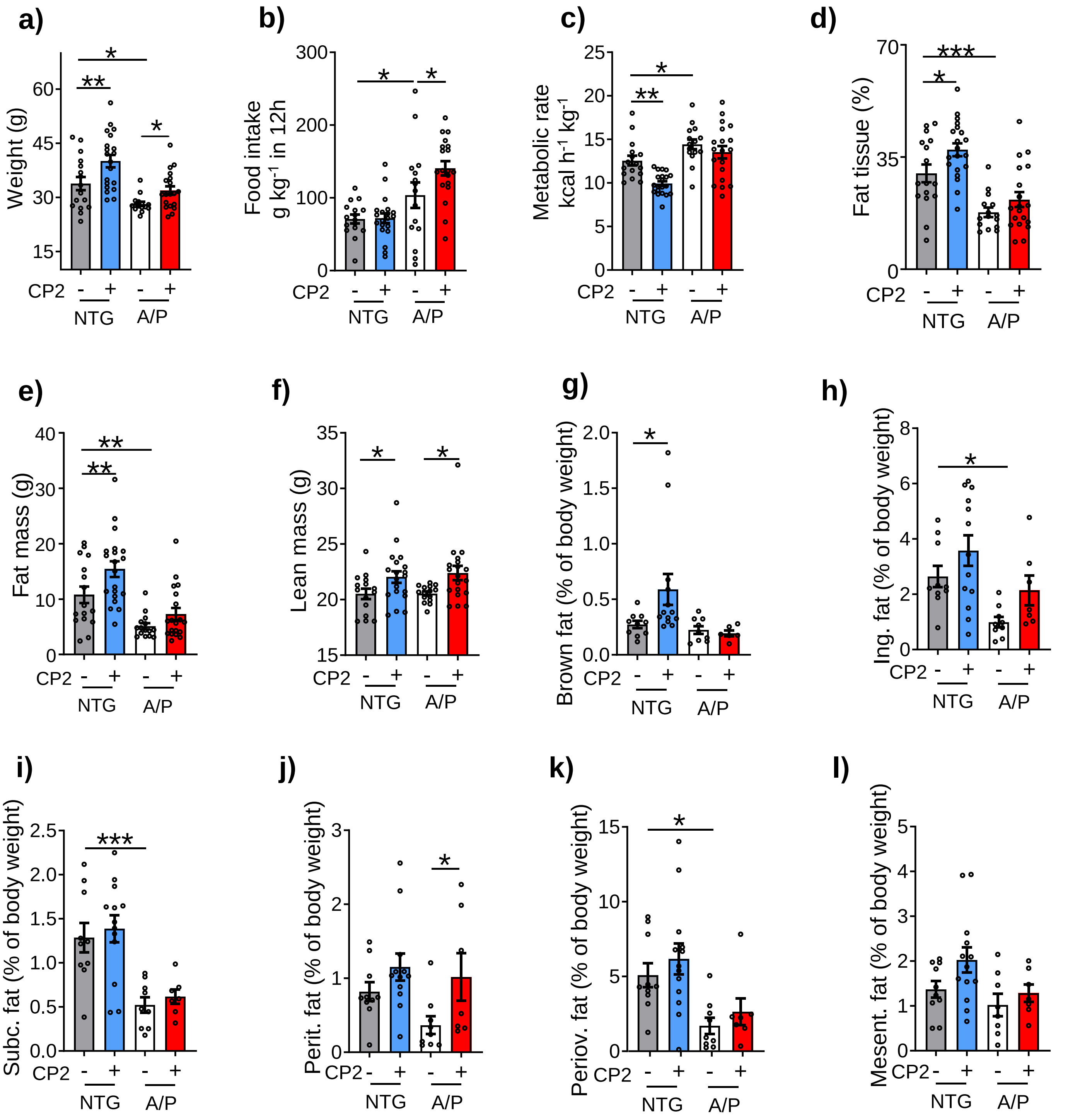

Supplementary Figure 3. CP2 treatment ameliorates body weight loss in symptomatic APP/PS1 mice. a) Body weight was significantly increased in CP2-treated NTG and APP/PS1 mice relative to untreated counterparts. Vehicle-treated APP/PS1 mice were significantly lighter compared to untreated NTG mice. CP2-treated APP/PS1 mice did not differ significantly from vehicle-treated NTG mice. **b)** Food intake was significantly increased in APP/PS1 mice in comparison to vehicle-treated NTG mice. CP2 treatment further increased food intake in APP/PS1 mice. **c)** Metabolic rate was increased in APP/PS1 compared to NTG mice. CP2 treatment decreased metabolic rate in NTG mice but did not have an effect in APP/PS1 mice. **d,e)** CP2 treatment significantly increased fat mass in NTG mice with a similar trend in APP/PS1 mice compared to vehicle-treated counterparts. **f)** CP2 treatment significantly increased lean mass in NTG and APP/PS1 mice. **g,h)** CP2 treatment increased brown fat in NTG mice (**g**), with a trend towards increasing inguinal fat in both NTG and APP/PS1 mice (**h**). **i)** CP2 treatment did not affect subcutaneous fat mass in APP/PS1 or NTG mice. **j,k)** CP2 treatment significantly increased the percentage of peritoneal fat in APP/PS1 mice (**j**) with a similar trend for periovarian fat in APP/PS1 and NTG mice (**k**). **l)** CP2 treatment did not affect the percentage of mesenteric fat in both NTG and APP/PS1 mice. All mice were 23-month-old treated with CP2 for 13 - 14 months. Significant changes were determined by two-way ANOVA with Fisher`s LSD *post-hoc* test. Data are mean ± S.E.M.; NTG, n = 16 mice *per* group; NTG+CP2, n = 20 mice *per* group; APP/PS1, n =15 mice *per* group; APP/PS1+CP2, n =19 mice *per* group. *P < 0.05, **P < 0.01, ***P < 0.001.

a)

	Dose mg kg^{-1}	T(1/2) h	Tmax h	Cmax ng ml^{-1}	AUC(0-24 h) ng ml^{-1}*h	AUC(0-inf) ng ml^{-1}*h	Cl/F ml h^{-1} kg-1	F (%)
IV_{pl}	3	6.03	0.08	4863.00	3655	3655	821	100
PO_{pl}	25	2.09	1.01	4512.00	19777	19785	1264	64.96

b)

Time (h)	CP2 [ng ml^{-1}] from IV
0.08	2009.60
0.08	2054.70
0.08	6683.70
0.25	2458.40
0.25	5557.70
0.27	3952.2
0.50	2549.5
0.50	2630.6
0.50	2436.8
1.00	809.8
1.00	767
1.02	767.8
2.08	403.8
2.03	411.8
2.00	231.4
2.02	235.5
4.00	42.402
4.00	47.161
4.00	35.961
8.00	3.342
8.00	3.034
8.00	4.616
24.00	0.682
24.00	0.549
24.00	0.517

c)

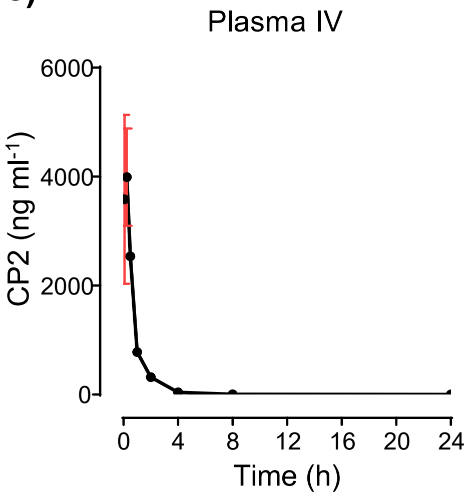

d)

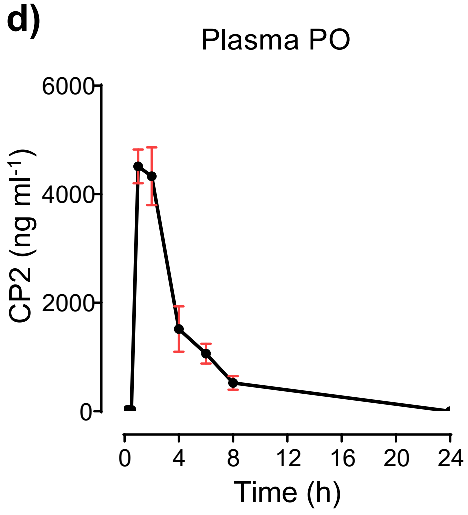

e)

Time (h)	Plasma [ng ml^{-1}] from PO
0.25	32.22
0.25	34.62
0.25	21.85
0.50	25.82
0.50	25.85
0.50	26.25
1.00	5026.3
1.00	4553.6
1.02	3956.1
1.98	3923.4
2.00	5388.2
2.00	3681.7
4.00	1108.6
4.02	1090.3
4.00	2351.5
6.05	883.8
6.00	878.1
5.98	1426.8
8.00	360.5
7.98	769.1
8.00	439.2
24.00	2.62
24.00	2.41
24.00	3.05

Supplementary Figure 4. Pharmacokinetics (PK) of CP2 delivered via oral (PO) or intravenous (IV) administration. a) Summary of PK study of CP2 delivered to C57BL/6 female mice (*n* = 3 *per* time point) via IV route (3 mg/kg) or *via* gavage (PO route) (25 mg/kg). The maximal plasma concentration following PO route was 4,512 ng/ml. The T1/2 values following drug administration in plasma were around 6 h and 2 h for IV and PO, respectively. The bioavailability (F) was estimated at 65 %. AUC, areas under the curve. **b,c)** CP2 levels in plasma after the IV administration (3 mg/kg). **d,e)** CP2 levels in plasma after the PO administration (25 mg/kg *via* gavage). Tmax, time to reach maximum (peak) plasma concentration following drug administration; Cmax, maximum (peak) plasma drug concentration; AUC (0-24 h), area under the plasma concentration-time curve over the last 24 h dosing interval; AUC (0-inf), area under the plasma concentration-time curve to infinity represents the total drug exposure across time; CI/F, apparent total clearance of the drug from plasma after oral administration.

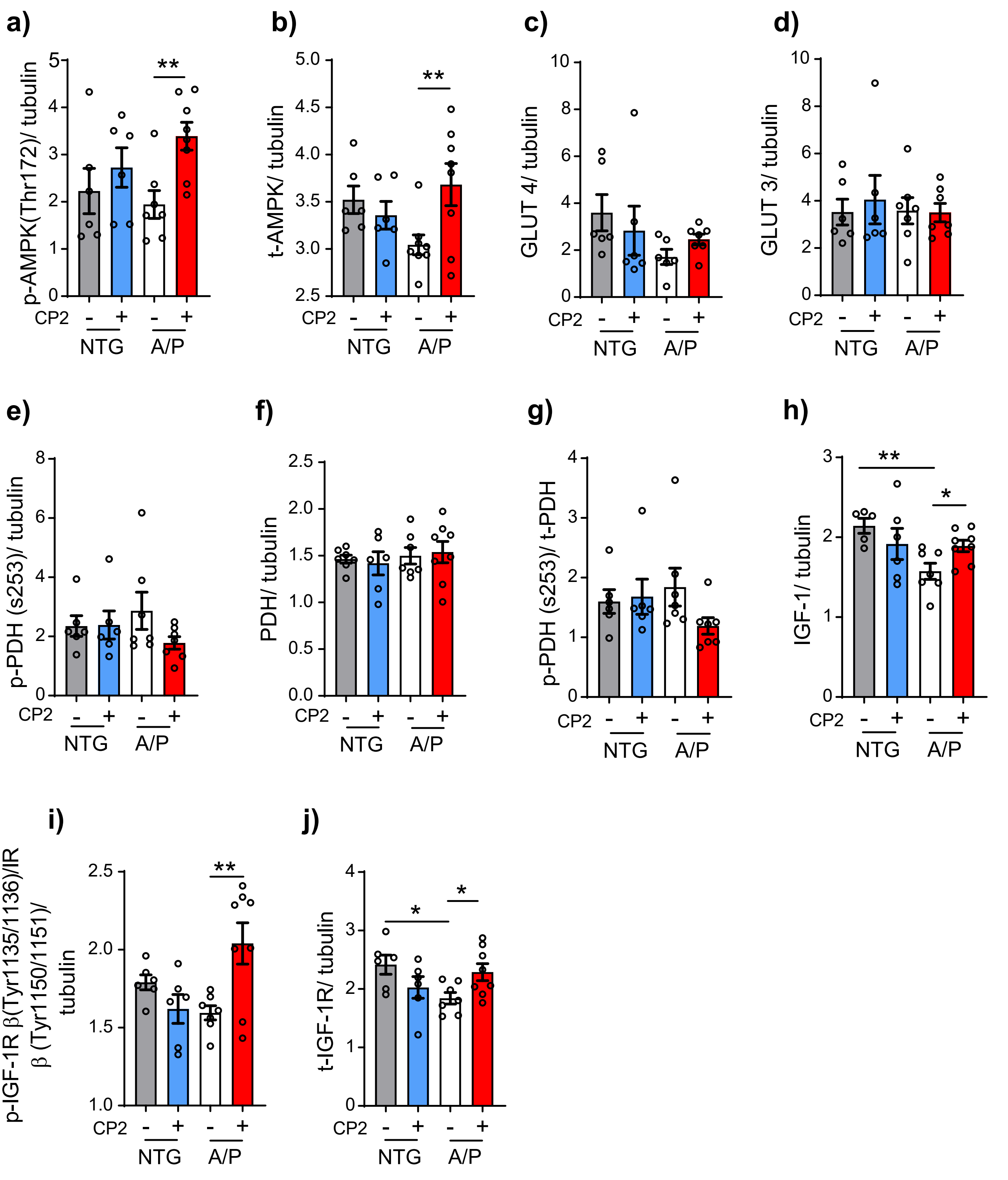

Supplementary Figure 5. CP2 treatment improves glucose uptake and utilization in APP/PS1 mice. a-j) The quantification of protein levels relevant to the IGF-1 signaling pathway in the brain tissue of CP2- and vehicle-treated APP/PS1 and NTG mice after 13 - 14 months of treatment using Western blot assay. Significance was determined by two-way ANOVA with Fisher`s LSD *post-hoc* test. Data are presented as mean ± S.E.M. *P < 0.05; **P < 0.01; NTG, *n* = 6 mice *per* group; NTG+CP2, *n* = 6 mice *per* group; APP/PS1, *n* = 8 mice *per* group; APP/PS1+CP2, *n* = 8 mice *per* group.

Western blots for Fig. 3i

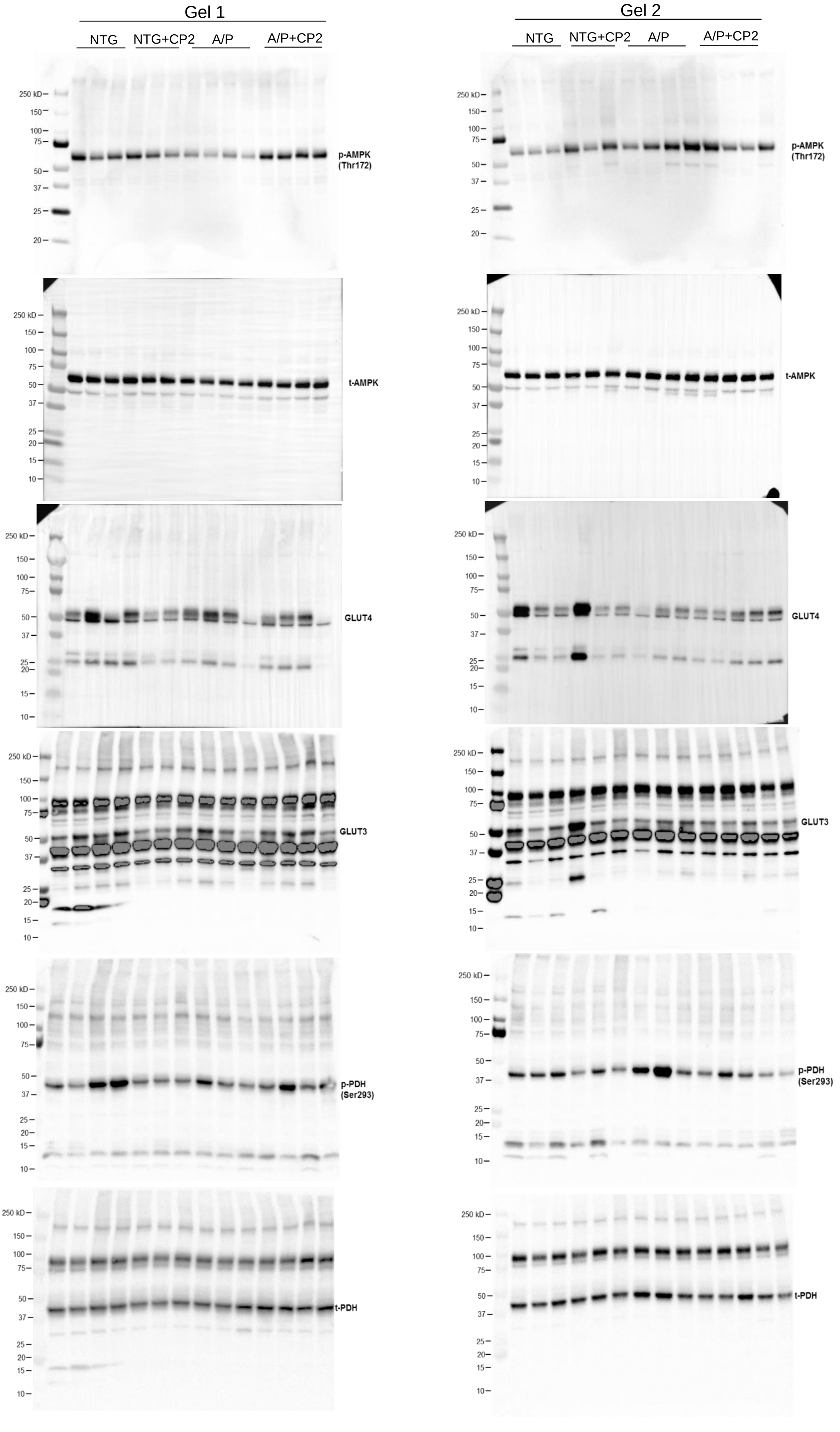

Western blots for Fig. 3i

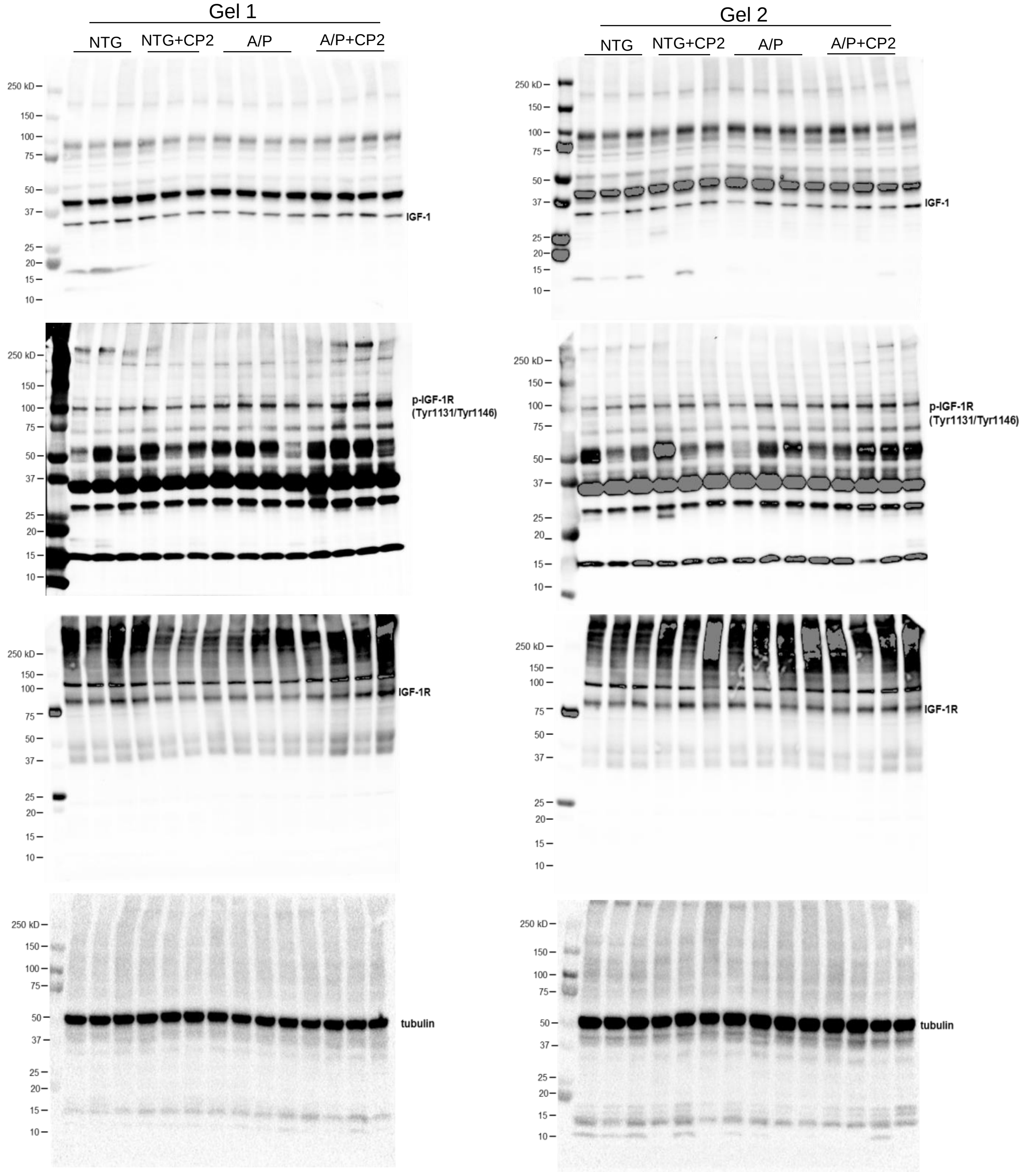

Supplementary Figure 6. Original uncropped Western blot images for Figure 3i. Cortico-hippocampal region of 6 - 8 mice *per* group was taken for Western blot analysis (NTG, *n* = 6; NTG+CP2, *n* = 6; APP/PS1, *n* = 8; APP/PS1+CP2, *n* = 8).

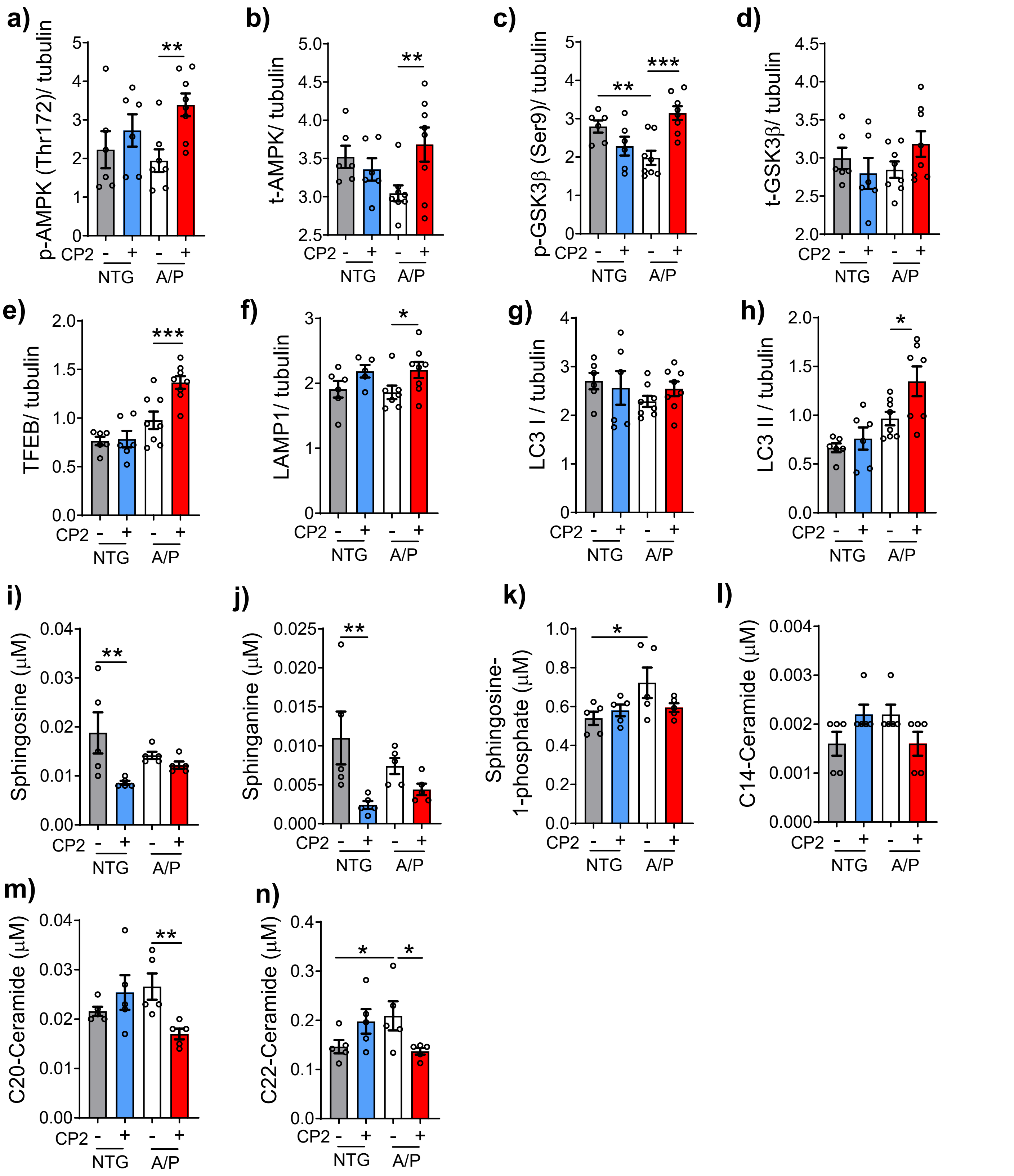

Supplementary Figure 7. CP2 treatment induces autophagy in the brain and reduces levels of plasma ceramides in symptomatic APP/PS1 mice. a-h) CP2-dependent activation of AMPK and inhibition of GSK3β activates TFEB and increases autophagy in CP2-treated APP/PS1 mice. Quantification of Western blot assays performed in the brain tissue of 23-month-old NTG and APP/PS1 mice treated with vehicle or CP2. NTG, $n = 6$ mice *per* group; NTG+CP2, $n = 6$ mice *per* group; APP/PS1, $n = 8$ mice *per* group; APP/PS1+CP2, $n = 8$ mice *per* group. **i-n)** Plasma from vehicle- and CP2-treated NTG and APP/PS1 mice 23-month-old ($n = 5$ *per* group) was obtained via retro-orbital bleeding. Ceramide concentrations were determined using targeted metabolomics approach and electrospray ionization mass spectrometry ESI/MS. Significance was determined by two-way ANOVA with Fisher`s LSD *post-hoc* test. Data are presented as mean ± S.E.M. *P< 0.05; **P < 0.01; ***P < 0.001.

Western blots for Fig. 4g

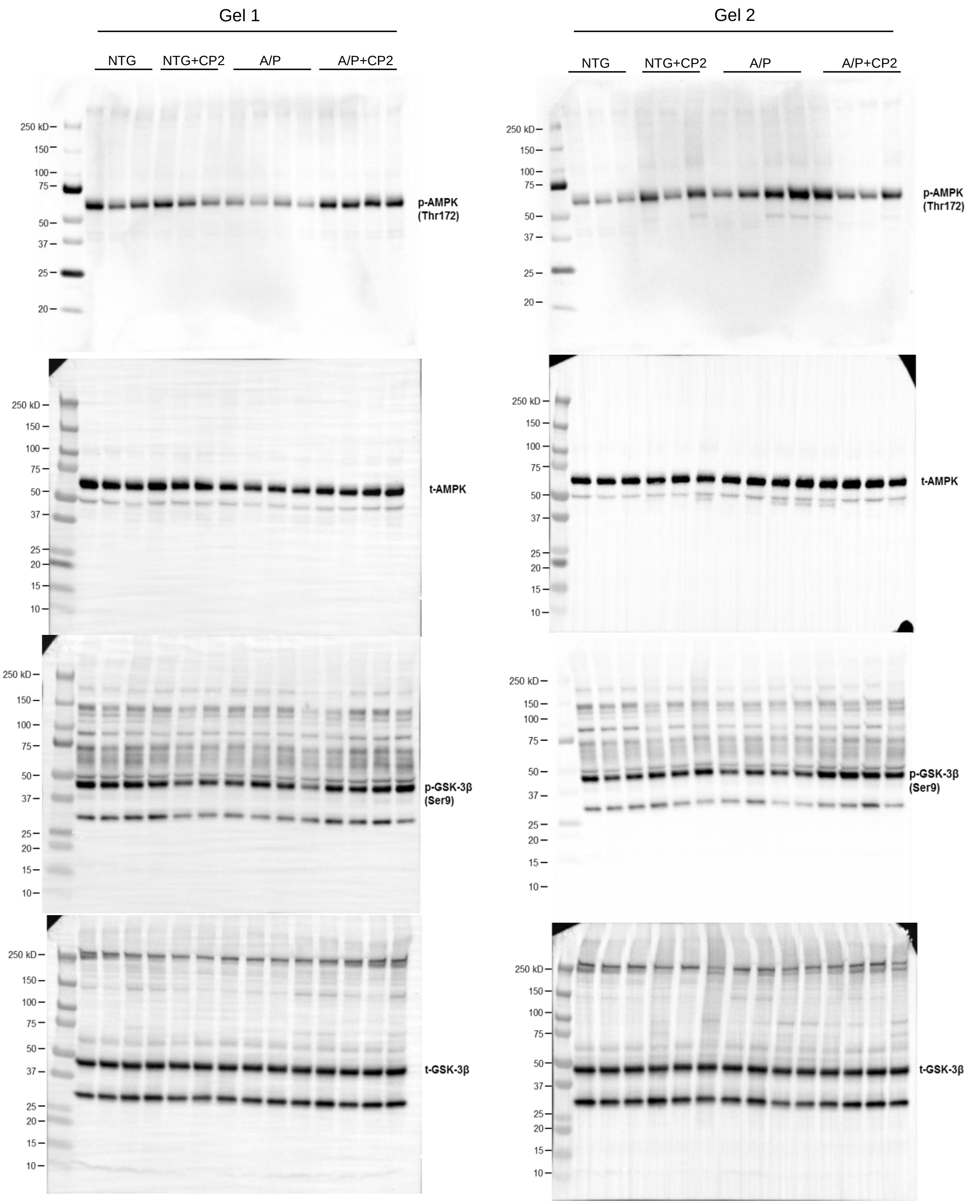

Western blots for Fig. 4g

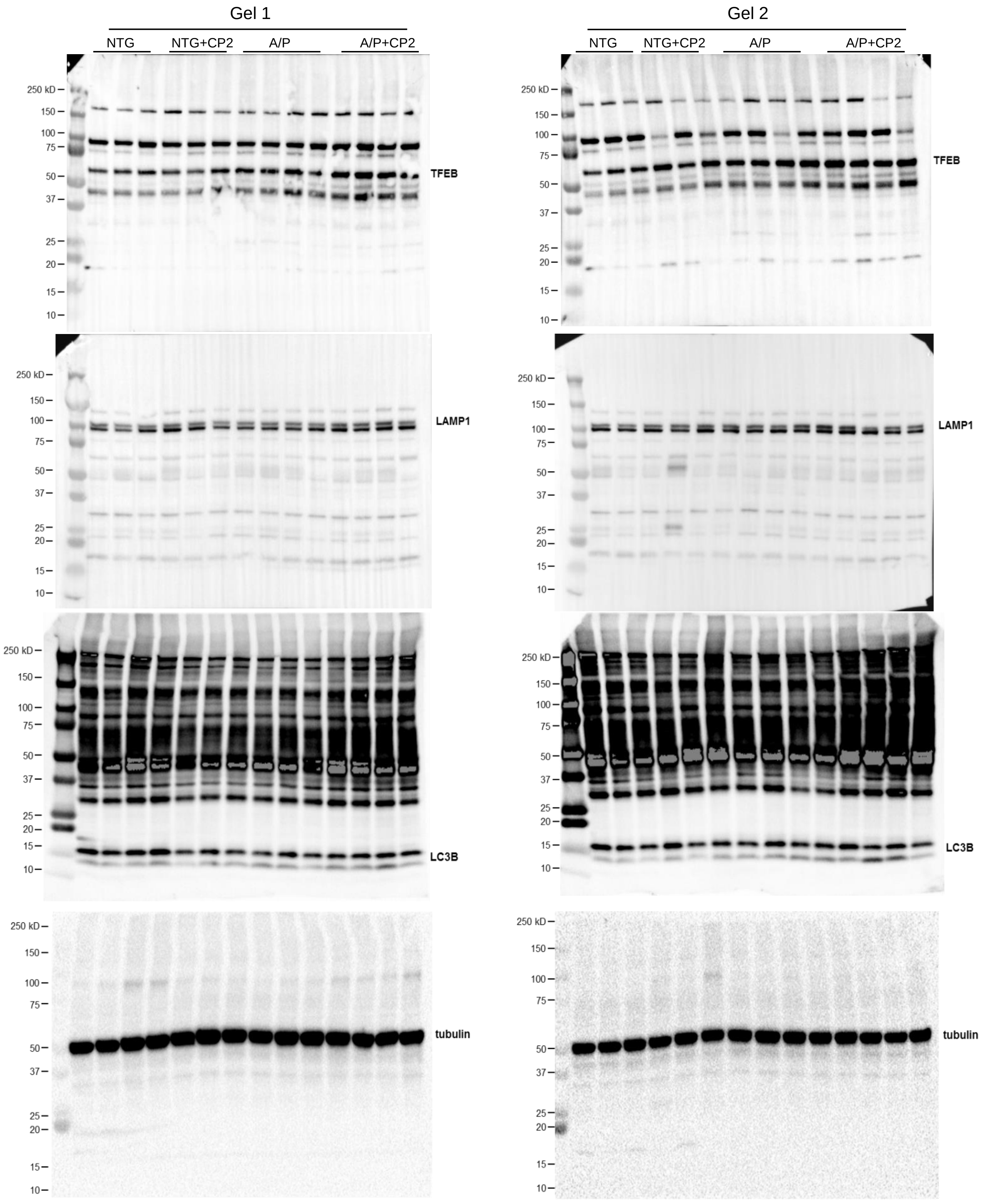

Supplementary Figure 8. Original uncropped Western blot images for Figure 4g. Cortico-hippocampal region of 6 - 8 mice *per* group was taken for Western blot analysis (NTG, *n* = 6; NTG+CP2, *n* = 6; APP/PS1, *n* = 8; APP/PS1 +CP2, *n* = 8).

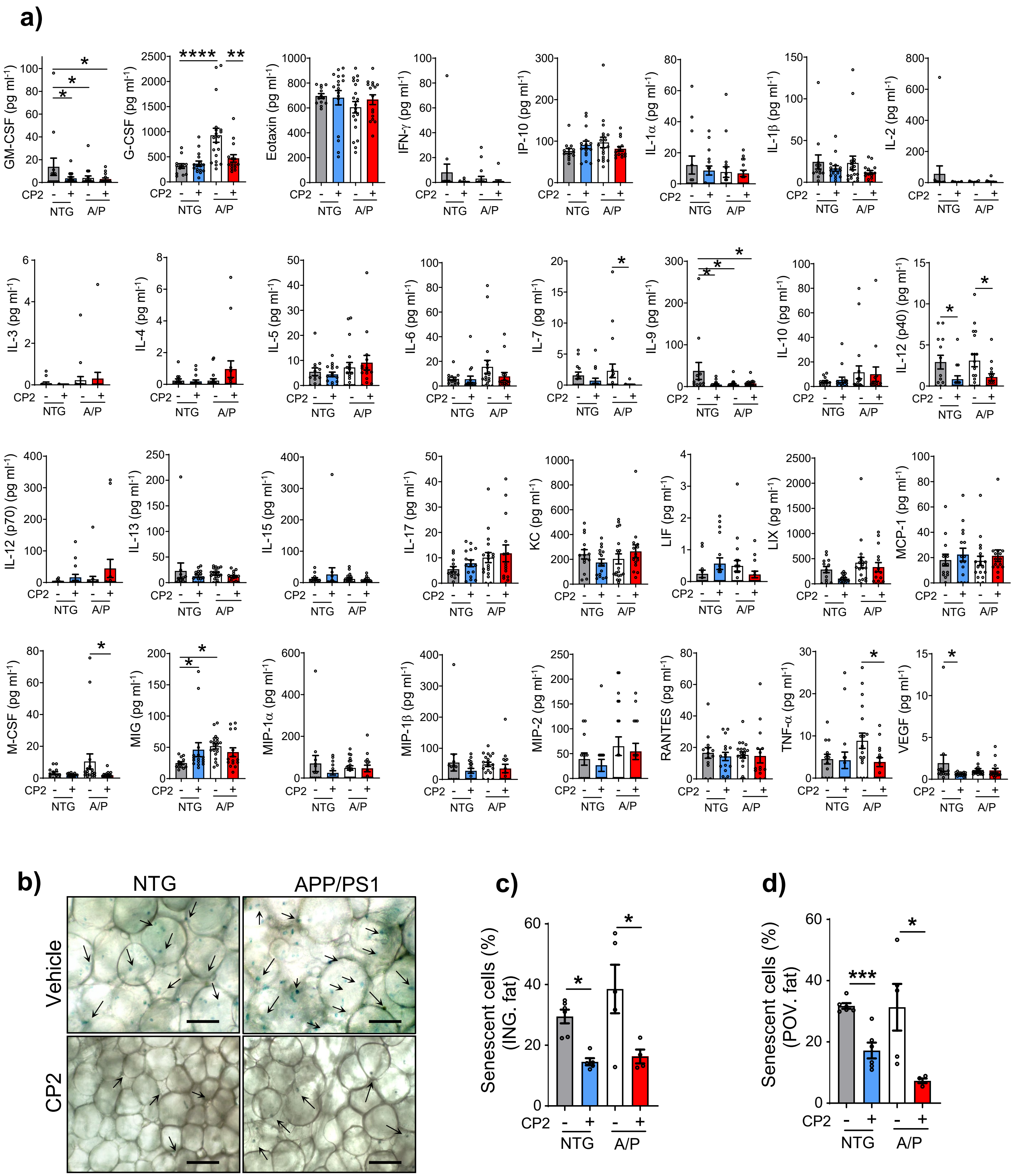

Supplementary Figure 9. CP2 treatment reduces inflammatory markers in blood and level of senescence cells in adipose tissue of symptomatic APP/PS1 and NTG mice. a) A panel of 32 cytokines and chemokines measured in plasma from 23-month-old symptomatic APP/PS1 and NTG vehicle- and CP2- treated mice. NTG, *n* = 13 mice *per* group; NTG+CP2, *n* = 17 mice *per* group; APP/PS1, *n* = 20 mice *per* group; APP/PS1+CP2, *n* = 16 mice *per* group. Plasma was obtained via retro-orbital bleeding; ELISA was conducted by Eve Technologies. **b)** Representative images of β-Gal staining of senescence cells in periovarian (POV) and inguinal (ING) adipose tissue from 23-month-old vehicle and CP2-treated NTG and APP/PS1 mice. Scale bar, 100 μm. **c,d)** Quantification of β-Gal staining in ING (**c**) and POV (**d**) fat depots from (**b**). NTG, *n* = 6 mice *per* group; NTG+CP2, *n* = 6 mice *per* group; APP/PS1, *n* = 5 mice *per* group; APP/PS1+CP2, *n* = 4 mice *per* group. A two-way ANOVA with Fisher`s LSD *post-hoc* test was used for statistical analysis. Data are presented as mean ± S.E.M. *P < 0.05; **P < 0.01; ****P < 0.0001.

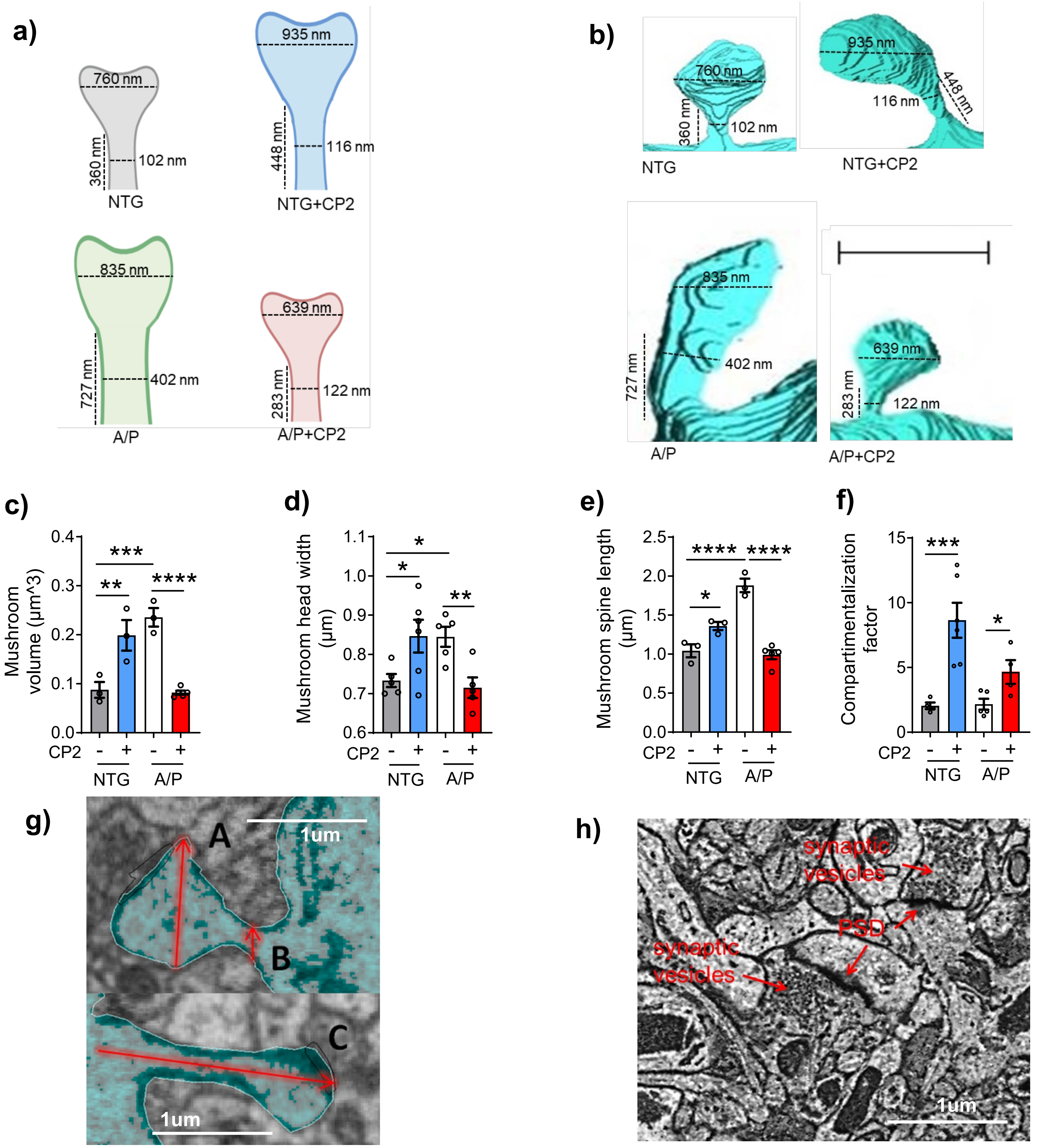

Supplementary Figure 10. CP2 treatment improves the morphology of dendritic spines required for LTP formation and synaptic function. **a)** Schematic illustration of morphological alterations in dendritic spines observed in CP2-treated NTG and APP/PS1 mice. Numbers indicate mean values. **b)** Representative images of mushroom spines generated using 3DEM from brain tissue of vehicle- and CP2-treated NTG and APP/PS1 mice. Scale bar, 1 µm. APP/PS1 mice have larger spine heads and longer and wider spine necks, which is associated with reduced ability to maintain LTP. **c-e)** Multiple parameters of dendritic spine morphology depicted in **(a,b)** measured in vehicle- and CP2-treated NTG and APP/PS1 mice including the volume of mushroom spines (**c**); mushroom head width (**d**); and mushroom spine length (**e**). Increased spine maturation after CP2 treatment is supported by measurements of the overall spine volume and spine geometry, especially the length and the width of the neck and the head, which are essential parameters influencing the ability of spines to retain synaptic receptors. **f)** The compartmentalization factor (CF), a measure of biochemical compartmentalization of spine synapses (see definition in the Method), calculated for each mushroom spine in vehicle *vs.* CP2-treated NTG and APP/PS1 mice. Significant increase in CF in CP2-treated mice indicates stronger ability to maintain LTP. **g)** Example of measurements of head diameter (A), neck width (B), and spine length from base to head (C). **h)** Representative EM micrograph of the CA1 hippocampal region utilized in the calculation of active synapses. Identification of synapses was done based on the presence of postsynaptic density (PSD) and synaptic vesicles. Scale bar, 1 µm. Data are presented as mean±S.E.M. A two-way ANOVA with Fisher`s LSD *post-hoc* test was used for statistical analysis. NTG, n = 5 mice *per* group; NTG+CP2, n = 6 mice *per* group; APP/PS1, n = 5 mice *per* group; APP/PS1+CP2, n = 5 mice *per* group.*P < 0.05; **P < 0.01; ***P<0.001; ****P<0.0001.

Western blots for Fig. 6e

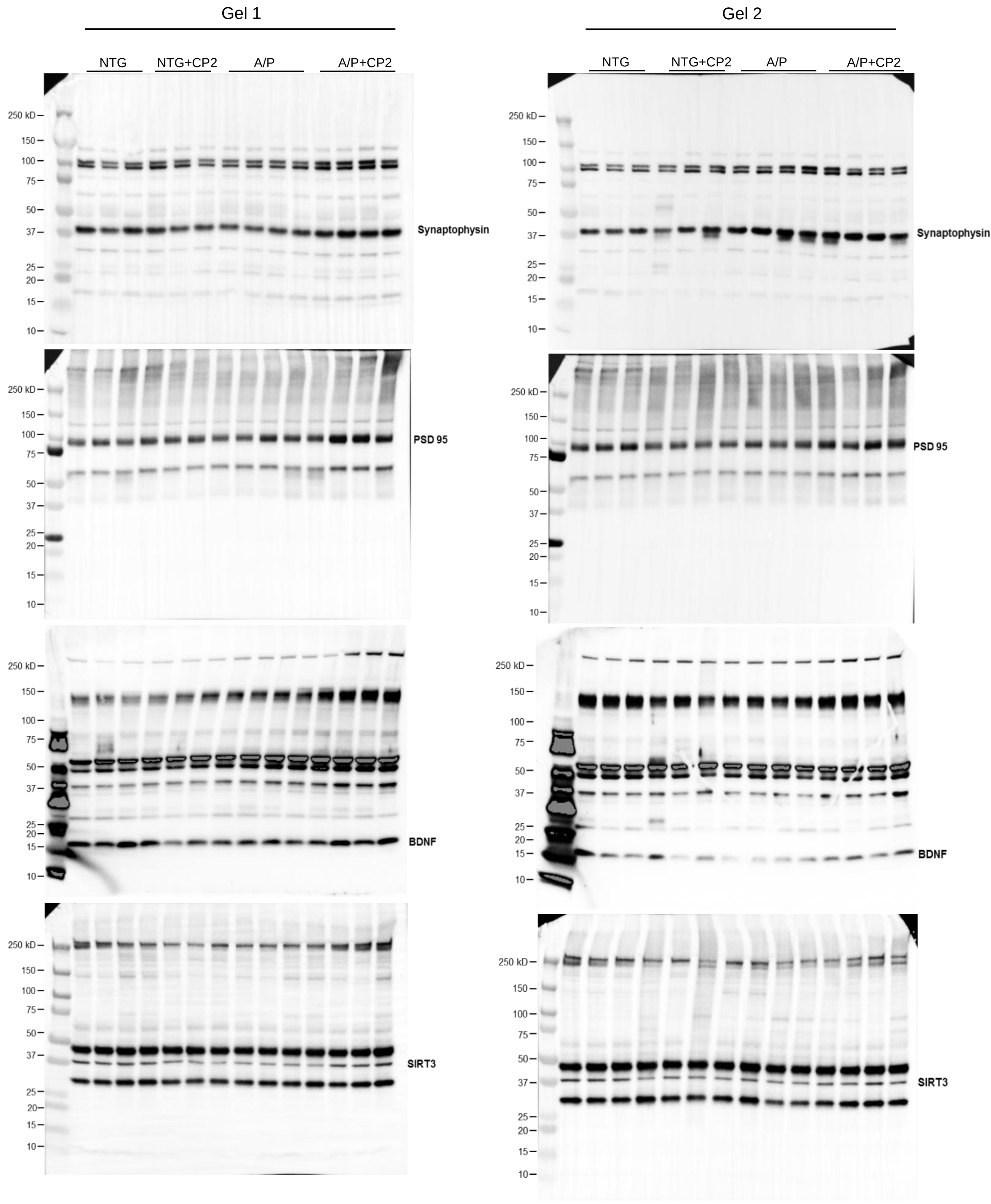

Western blots for Fig. 6e

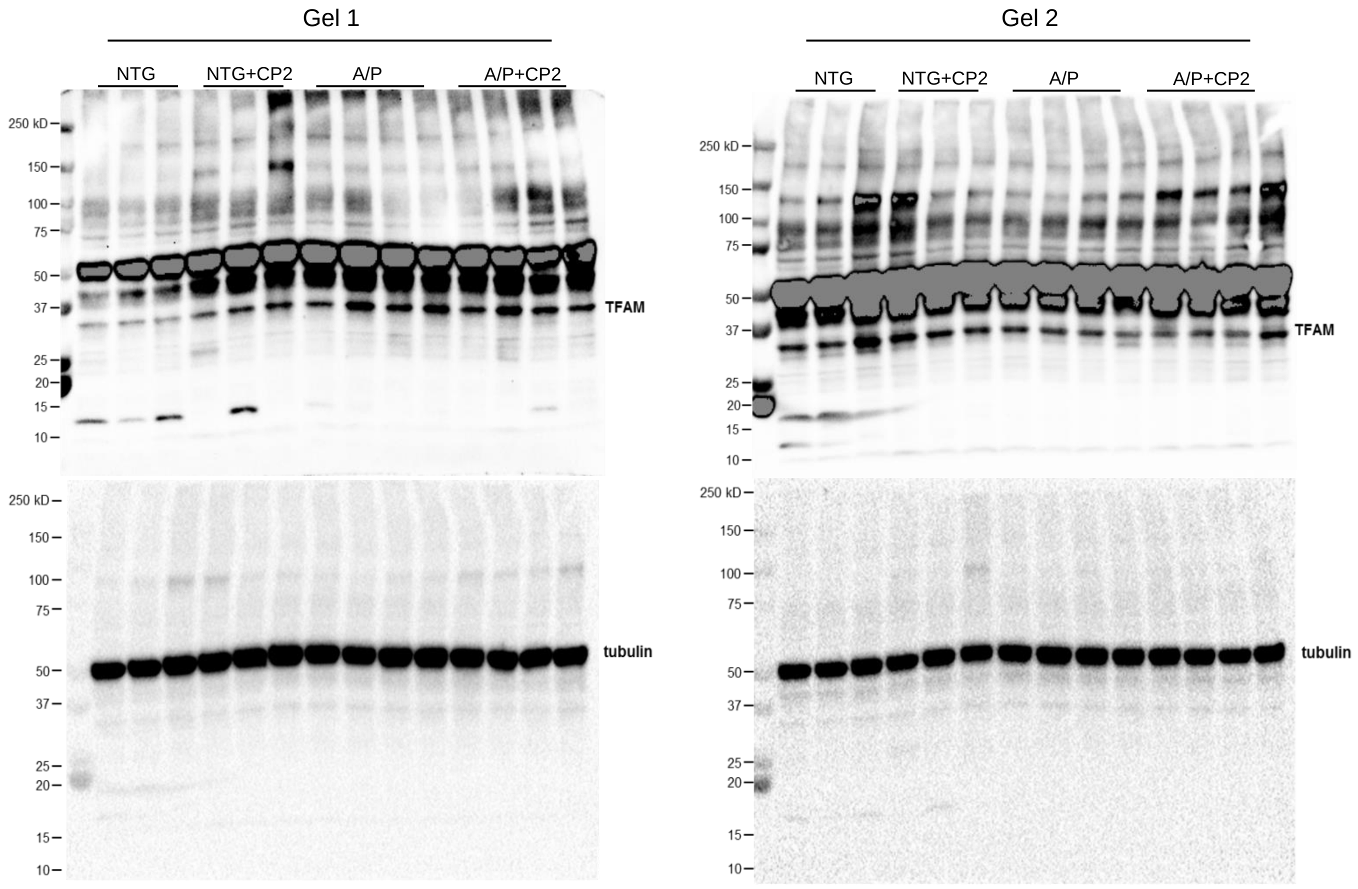

Supplementary Figure 11. Original uncropped Western blot images for Figure 6k. Cortico-hippocampal region of 6 - 8 mice *per* group was taken for Western blot analysis (NTG, n = 6; NTG+CP2, n = 6; APP/PS1, n = 8; APP/PS1+CP2, n = 8).

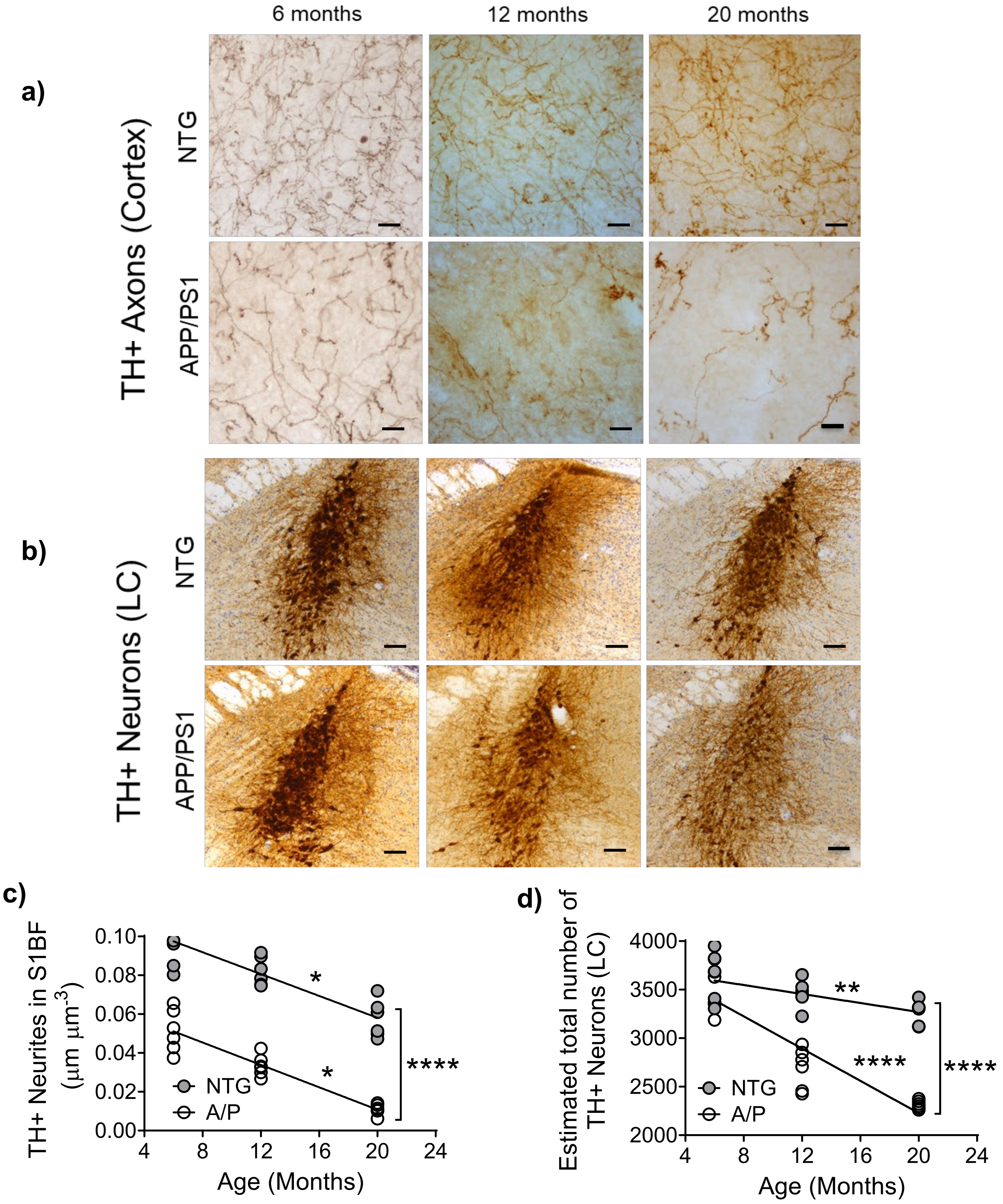

Supplementary Figure 12. APP/PS1 mice display progressive neurodegeneration of TH+ neurons in cortex and *locus coeruleus* (LC). NTG and APP/PS1 mice were evaluated for the integrity of TH+ axons in S1BF (**a,c**) and TH+ neurons in LC (**b,d**) at 6, 12, and 20 months of age. Representative images of TH+ axons in S1BF (**a**) and TH+ neurons in LC (**b**). **c)** Progressive loss of TH+ axons was observed in both APP/PS1 (R squared = 0.9997, P = 0.0102) and NTG mice (R squared = 0.9952, P = 0.0442). The rate of age-related decline of TH+ axons was similar between NTG and APP/PS1 (differences between slopes are not significant P = 0.7226), but magnitude of TH+ axonal loss was greater in APP/PS1 mice (differences between the slopes elevation are significant P < 0.0001). **d)** The age-related loss of TH+ neurons was observed starting from 6 months of age in both NTG (R squared = 0.3733, P = 0.0071) and APP/PS1 mice (R squared = 0.8231, P < 0.0001), and additionally APP/PS1 mice showed greater age-related loss of TH+ neurons (P < 0.0001). *n* = 6 female mice *per* group in each test. Data are represented as mean±S.E.M. A linear regression analysis was applied for statistical comparison between the groups. **P < 0.01, ***P < 0.001, ****P < 0.0001. Scale bars: a, 20 μm; b, 100 μm.

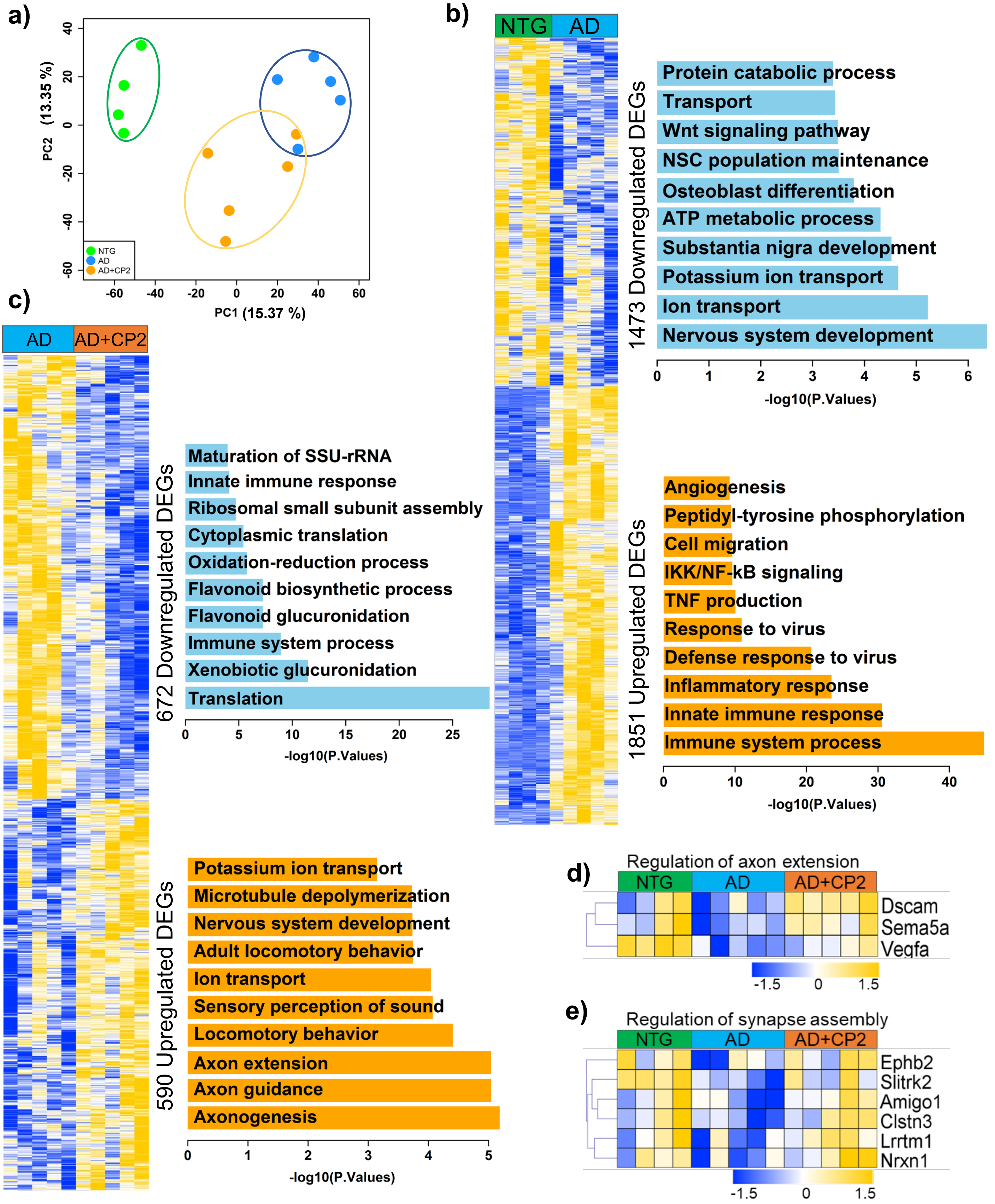

Supplementary Figure 13. Transcriptomic changes in brain tissue from vehicle and CP2-treated NTG and APP/PS1 mice determined using RNA-seq. a) Principal Component Analysis (PCA) shows separated clusters of samples among 3 groups (NTG, green; APP/PS1, blue; APP/PS1+CP2, orange) where CP2 treatment produces specific changes allowing group separation. Variances explained by the first two principal components are shown here (15.37% and 13.35%, respectively). **b)** DEGs in NTG vs. APP/PS1 mice and function enrichment analysis based on up- or down-regulated genes. **c)** DEGs in APP/PS1 vs. APP/PS1+CP2 mice and function enrichment analysis based on up- or down-regulated genes. **d)** A heatmap shows changes in genes of pathway related to the positive regulation of axon extension, which were up-regulated after CP2 treatment in APP/PS1 mice. **e)** A heatmap shows changes in genes involved in synapse assembly that were up-regulated by CP2 treatment in APP/PS1 mice. All mice were 23 months of age treated with CP2 or vehicle for 13-14 months. NTG, $n = 4$ mice *per* group; APP/PS1, $n = 5$ mice *per* group; APP/PS1+CP2, $n = 5$ mice *per* group.

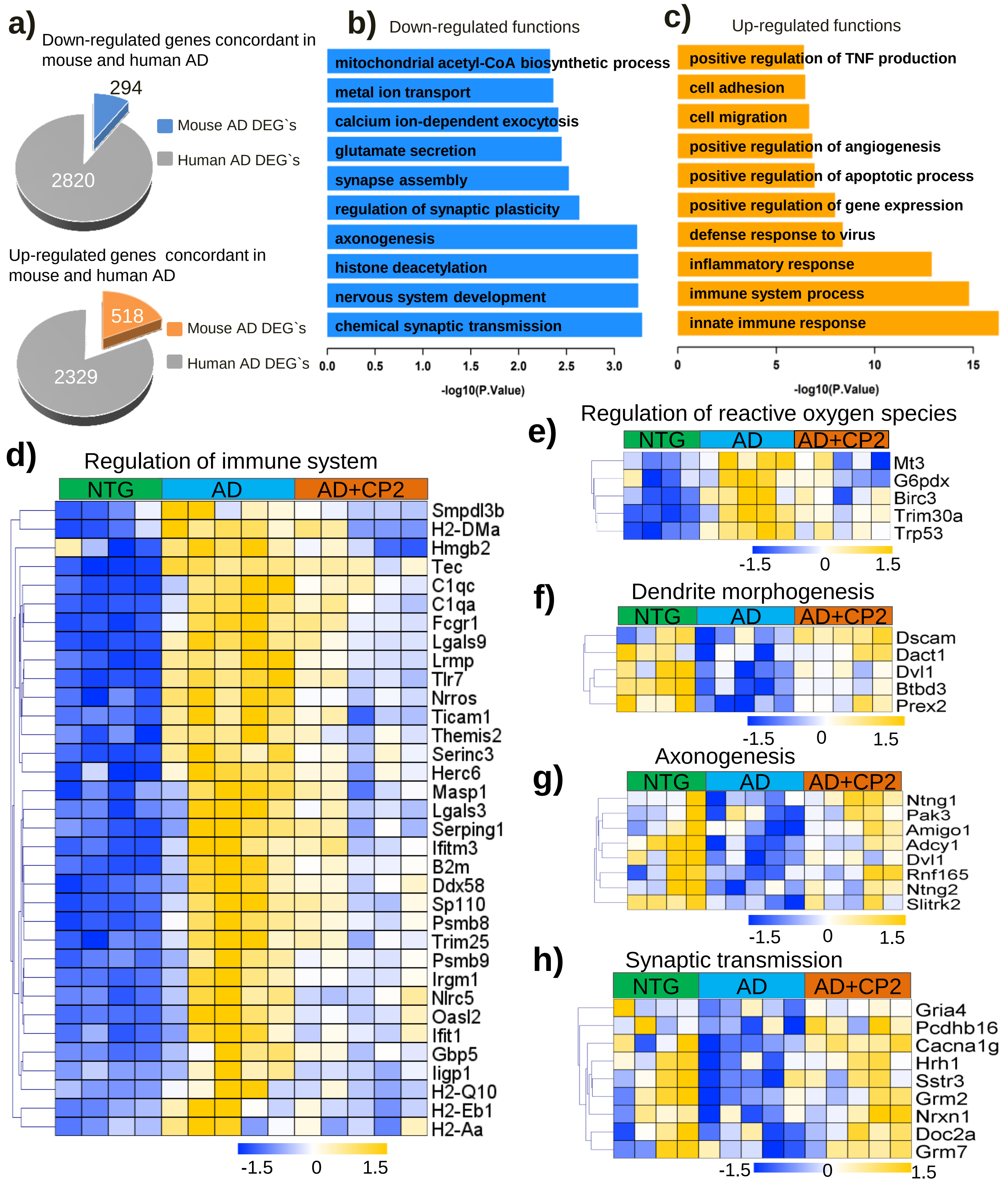

Supplementary Figure 14. CP2 treatment affects pathways relevant to AD patients based on the cross-validation of mouse and human AMP-AD transcriptomic data. a) Comparison of human DEGs from the AMP-AD set with 567 DEGs shown in Figure 8a that are specifically affected by CP2 treatment in APP/PS1 mice. **b)** Functions identified by the enrichment analysis associated with the up-regulated DEGs (567) in APP/PS1 mice mapped against human up-regulated AD DEGs. These functions were reversed (down-regulated) by CP2 treatment in APP/PS1 mice. **c)** Functions identified by the enrichment analysis associated with the down-regulated DEGs (567) in APP/PS1 mice mapped with human down-regulated AD DEGs. These functions were reversed (up-regulated) by CP2 treatment in APP/PS1 mice. **d, e)** Heatmaps of changes in genes associated with immune system (**d**) and the reactive oxygen species (**e**) were down-regulated after CP2 treatment in APP/PS1 mice. **f-h)** Heatmaps of changes in genes associated with dendrite morphogenesis (**f**), axonogenesis (**g**), and synaptic transmission (**h**) were up-regulated after CP2 treatment in APP/PS1 mice. The weight of the edges corresponds to the confidence scores of gene integration. All mice were 23-month-old treated with CP2 or vehicle for 13-14 months. NTG, *n* = 4 mice *per* group; APP/PS1, *n* = 5 mice per group; APP/PS1 +CP2, *n* = 5 *per* group.

Supplementary Table 1. CP2 levels in the brain tissue and plasma of NTG and APP/PS1 mice 22 – 24-month-old chronically treated with CP2 from 9 months of age[a].

Genotype	Brain (ng g^{-1})	Brain (nM)	Plasma (ng ml^{-1})	Plasma (nM)
NTG	24.77	62.96	44.44	112.95
NTG	16.91	42.98	138.32	351.56
NTG	14.57	37.03	40.95	104.08
NTG	14.44	36.70	138.28	351.46
NTG	52.22	132.72	419.32	1,065.75
NTG	110.37	280.52	66.7	169.53
NTG	9.97	25.34	21.96	55.81
APP/PS1	7.34	18.66	187.06	475.44
APP/PS1	44.37	112.77	349.88	889.26
APP/PS1	4.52	11.49	67.44	171.41
APP/PS1	10.36	26.33	161.3	409.96
APP/PS1	50.23	127.67	89.664	227.89
APP/PS1	16.36	41.58	174.72	444.07
APP/PS1	11.99	30.47	32.42	82.40
APP/PS1	16.37	41.61	28.31	71.95
APP/PS1	14.23	36.17	10.69	27.17
APP/PS1	9.15	23.26	67.22	170.85
APP/PS1	11.51	29.25	52.76	134.10

[a] CP2 levels were measured in cerebellum. Blood for this analysis was collected at the end of the study at the time of sacrifice.

Supplementary Table 2. Results of kinome profiling for CP2 in the Nanosyn 250 Kinase panel. The number represents % inhibition at the concentration tested. Negative numbers represent increased enzyme activity. Numbers below 20 considered to be within the acceptable noise level within the plate.

Kinase	Conc. Tested (μM)	CP2
ABL1	1	-4
ABL1	10	4
AKT1	1	3
AKT1	10	6
AKT2	1	1
AKT2	10	-4
AKT3	1	0
AKT3	10	1
ALK	1	2
ALK	10	-2
AMP-A1B1G1	1	-3
AMP-A1B1G1	10	-13
AMP-A2B1G1	1	3
AMP-A2B1G1	10	1
ARG	1	-1
ARG	10	2
ARK5	1	-3
ARK5	10	17
AURORA-A	1	3
AURORA-A	10	-4
AURORA-B	1	1
AURORA-B	10	7
AURORA-C	1	3
AURORA-C	10	-7
AXL	1	2
AXL	10	-7
BLK	1	36
BLK	10	34
BMX	1	3
BMX	10	-6
BRAF	1	0
BRAF	10	-4
BRK	1	-3
BRK	10	-15
BRSK1	1	-2
BRSK1	10	-4
BRSK2	1	-10
BRSK2	10	-14
BTK	1	1
BTK	10	1
CAMK1A	1	2
CAMK1A	10	1
CAMK1D	1	2
CAMK1D	10	-4
CAMK2A	1	-1
CAMK2A	10	0
CAMK2B	1	1
CAMK2B	10	4
CAMK2D	1	-2
CAMK2D	10	-1
CAMK2G	1	-5
CAMK2G	10	-7
CAMK4	1	0
CAMK4	10	-6
CDK1	1	-4
CDK1	10	0
CDK2	1	-3
CDK2	10	-4
CDK2-CYCLINE	1	4
CDK2-CYCLINE	10	1
CDK3-CYCLINE	1	1
CDK3-CYCLINE	10	-2
CDK4-CYCLIND	1	-1
CDK4-CYCLIND	10	3
CDK5	1	0
CDK5	10	3
CDK5-P25	1	8
CDK5-P25	10	2
CDK6-CYCLIND3	1	0
CDK6-CYCLIND3	10	-3
CDK7	1	4
CDK7	10	11
CDK9-CYCLINT1	1	5
CDK9-CYCLINT1	10	-26
CHEK1	1	-1
CHEK1	10	-10
CHEK2	1	1
CHEK2	10	-1
CK1	1	1
CK1	10	19
CK1-EPSILON	1	2
CK1-EPSILON	10	30
CK1-GAMMA1	1	1
CK1-GAMMA1	10	19
CK1-GAMMA2	1	-3
CK1-GAMMA2	10	10
CK1-GAMMA3	1	1
CK1-GAMMA3	10	15
CLK1	1	-6
CLK1	10	-6
CLK2	1	-2
CLK2	10	3
CLK3	1	1
CLK3	10	-2
CLK4	1	5
CLK4	10	24
CRAF	1	-1
CRAF	10	0
CSK	1	0
CSK	10	-5
DAPK1	1	0
DAPK1	10	-1
DAPK3	1	-2
DAPK3	10	0
DCAMKL2	1	-2
DCAMKL2	10	-5
DDR1	1	3
DDR1	10	16

Supplementary Table 3.

Kinase	Conc. Tested (μM)	CP2
DDR2	1	4
DDR2	10	2
DYRK1A	1	4
DYRK1A	10	3
DYRK1B	1	2
DYRK1B	10	6
DYRK2	1	-7
DYRK2	10	1
DYRK3	1	1
DYRK3	10	2
DYRK4	1	-2
DYRK4	10	-3
EGFR	1	5
EGFR	10	-12
EPH-A1	1	-1
EPH-A1	10	-9
EPH-A2	1	1
EPH-A2	10	0
EPH-A3	1	-2
EPH-A3	10	-14
EPH-A4	1	0
EPH-A4	10	1
EPH-A5	1	-3
EPH-A5	10	-10
EPH-A8	1	0
EPH-A8	10	-9
EPH-B1	1	-3
EPH-B1	10	-12
EPH-B2	1	-3
EPH-B2	10	2
EPH-B3	1	-3
EPH-B3	10	-10
EPH-B4	1	-4
EPH-B4	10	-12
ERB-B2	1	-6
ERB-B2	10	-13
ERB-B4	1	8
ERB-B4	10	-1
FAK	1	0
FAK	10	-3
FER	1	-1
FER	10	1
FES	1	5
FES	10	-3
FGFR1	1	5
FGFR1	10	-15
FGFR2	1	1
FGFR2	10	-3
FGFR4	1	0
FGFR4	10	-4
FGR	1	0
FGR	10	-38
FLT-1	1	4
FLT-1	10	-23
FLT-3	1	0
FLT-3	10	0
FLT-4	1	1
FLT-4	10	2
FMS	1	4
FMS	10	-1
FRAP1	1	2
FRAP1	10	22
FYN	1	1
FYN	10	5
GRK6	1	-2
GRK6	10	-5
GRK7	1	0
GRK7	10	-12
GSK-3-ALPHA	1	5
GSK-3-ALPHA	10	47
GSK-3-BETA	1	-4
GSK-3-BETA	10	21
HASPIN	1	2
HASPIN	10	-1
HCK	1	9
HCK	10	-1
HIPK1	1	-6
HIPK1	10	2
HIPK2	1	0
HIPK2	10	0
HIPK3	1	-3
HIPK3	10	-1
HIPK4	1	-2
HIPK4	10	-4
IGF1R	1	4
IGF1R	10	-4
IKK-ALPHA	1	0
IKK-ALPHA	10	-3
IKK-BETA	1	0
IKK-BETA	10	6
IKK-EPSILON	1	-1
IKK-EPSILON	10	1
INSR	1	-1
INSR	10	1
IRAK1	1	-4
IRAK1	10	-4
IRAK4	1	0
IRAK4	10	3
IRR	1	4
IRR	10	-1
ITK	1	4
ITK	10	-3
JAK1	1	-2
JAK1	10	2
JAK2	1	4
JAK2	10	0
JAK3	1	-2
JAK3	10	-11
JNK1	1	2
JNK1	10	6
JNK2	1	6
JNK2	10	-3
JNK3	1	4
JNK3	10	9
KDR	1	6
KDR	10	-3

Supplementary Table 3.

Kinase	Conc. Tested (μM)	CP2
KIT	1	0
KIT	10	-6
LATS1	1	0
LATS1	10	-9
LATS2	1	0
LATS2	10	0
LCK	1	2
LCK	10	-10
LOK	1	4
LOK	10	14
LRRK2-G2019S	1	0
LRRK2-G2019S	10	3
LTK	1	2
LTK	10	-6
LYNA	1	1
LYNA	10	-2
LYNB	1	2
LYNB	10	-18
MAP4K2	1	3
MAP4K2	10	2
MAP4K4	1	4
MAP4K4	10	1
MAP4K5	1	1
MAP4K5	10	4
MAPK1	1	-1
MAPK1	10	-10
MAPK3	1	-1
MAPK3	10	3
MAPKAPK-2	1	-4
MAPKAPK-2	10	2
MAPKAPK-3	1	-1
MAPKAPK-3	10	-3
MARK1	1	2
MARK1	10	3
MARK3	1	-4
MARK3	10	-4
MARK4	1	-3
MARK4	10	0
MEK1	1	2
MEK1	10	-1
MEK2	1	-1
MEK2	10	6
MEK3	1	0
MEK3	10	2
MELK	1	6
MELK	10	3
MER	1	2
MER	10	-1
MET	1	8
MET	10	-9
MINK	1	0
MINK	10	10
MKNK1	1	0
MKNK1	10	5
MNK2	1	5
MNK2	10	5
MRCK-ALPHA	1	-2
MRCK-ALPHA	10	-3
MRCK-BETA	1	0
MRCK-BETA	10	-2
MSK1	1	-1
MSK1	10	-13
MSK2	1	1
MSK2	10	-11
MSSK1	1	-3
MSSK1	10	2
MST1	1	3
MST1	10	22
MST2	1	5
MST2	10	10
MST3	1	-2
MST3	10	-3
MST4	1	-2
MST4	10	-5
MUSK	1	4
MUSK	10	-6
NDR2	1	1
NDR2	10	-4
NDRG1	1	0
NDRG1	10	-2
NEK1	1	3
NEK1	10	7
NEK2	1	0
NEK2	10	-1
NEK6	1	4
NEK6	10	1
NEK7	1	3
NEK7	10	-20
NEK9	1	2
NEK9	10	5
P38-ALPHA	1	4
P38-ALPHA	10	1
P38-BETA	1	3
P38-BETA	10	1
P38-DELTA	1	0
P38-DELTA	10	-2
P38-GAMMA	1	0
P38-GAMMA	10	-7
P70S6K1	1	3
P70S6K1	10	-1
P70S6K2	1	1
P70S6K2	10	-14
PAK1	1	0
PAK1	10	2
PAK2	1	0
PAK2	10	-2
PAK3	1	-1
PAK3	10	-3
PAK4	1	5
PAK4	10	10
PAK5	1	1
PAK5	10	3
PAK6	1	4
PAK6	10	-3
PAR-1B-ALPHA	1	-3
PAR-1B-ALPHA	10	7

Supplementary Table 3.

Kinase	Conc. Tested (µM)	CP2
PASK	1	-5
PASK	10	2
PDGFR-ALPHA	1	3
PDGFR-ALPHA	10	3
PDGFR-BETA	1	4
PDGFR-BETA	10	-1
PDK1	1	-4
PDK1	10	0
PERK	1	2
PERK	10	-14
PHK-GAMMA1	1	3
PHK-GAMMA1	10	0
PHK-GAMMA2	1	1
PHK-GAMMA2	10	5
PI3-KINASE-ALPHA	1	2
PI3-KINASE-ALPHA	10	7
PI4-K-BETA	1	0
PI4-K-BETA	10	-3
PIM-1-KINASE	1	0
PIM-1-KINASE	10	1
PIM2	1	1
PIM2	10	-2
PIM3	1	-1
PIM3	10	0
PKA	1	2
PKA	10	-11
PKACB	1	-1
PKACB	10	0

Kinase	Conc. Tested (µM)	CP2
PKC-ALPHA	1	1
PKC-ALPHA	10	0
PKC-BETA1	1	0
PKC-BETA1	10	8
PKC-BETA2	1	2
PKC-BETA2	10	0
PKC-ETA	1	-2
PKC-ETA	10	1
PKC-GAMMA	1	2
PKC-GAMMA	10	1
PKC-IOTA	1	-6
PKC-IOTA	10	3
PKC-THETA	1	-3
PKC-THETA	10	-1
PKC-ZETA	1	-1
PKC-ZETA	10	-3
PKN1	1	-1
PKN1	10	-4
PKN2	1	2
PKN2	10	1
PLK1	1	-1
PLK1	10	-19
PLK3	1	-5
PLK3	10	14
PLK4	1	-9
PLK4	10	-11
PRAK	1	-3
PRAK	10	-8
PRKACA	1	-2

Kinase	Conc. Tested (µM)	CP2
PRKACA	10	-2
PRKD1	1	7
PRKD1	10	-4
PRKD2	1	0
PRKD2	10	2
PRKD3	1	5
PRKD3	10	-3
PRKG1	1	2
PRKG1	10	4
PRKX	1	0
PRKX	10	-1
PTK5	1	-1
PTK5	10	-3
PYK2	1	5
PYK2	10	-8
RET	1	3
RET	10	-4
RIPK2	1	0
RIPK2	10	-5
ROCK1	1	5
ROCK1	10	-1
ROCK2	1	7
ROCK2	10	16
RON	1	8
RON	10	-12
ROS	1	4
ROS	10	-5
RSK1	1	2
RSK1	10	0

Kinase	Conc. Tested (µM)	CP2
RSK2	1	-2
RSK2	10	-6
RSK3	1	-3
RSK3	10	-1
RSK4	1	-1
RSK4	10	-1
SGK1	1	1
SGK1	10	1
SGK2	1	-4
SGK2	10	1
SGK3	1	5
SGK3	10	-5
SIK	1	4
SIK	10	1
SLK	1	-2
SLK	10	4
SNF1LK2	1	4
SNF1LK2	10	1
SPHK1	1	3
SPHK1	10	0
SPHK2	1	4
SPHK2	10	-4
SRC	1	2
SRC	10	-18
SRMS	1	-6
SRMS	10	-10
SRPK1	1	3
SRPK1	10	-8
SRPK2	1	-3

Supplementary Table 3.

Kinase	Conc. Tested (μM)	CP2
SRPK2	10	0
STK16	1	1
STK16	10	-5
STK25	1	-1
STK25	10	2
SYK	1	6
SYK	10	-15
TAK1-TAB1	1	-1
TAK1-TAB1	10	-1
TAOK2	1	-2
TAOK2	10	-1
TAOK3	1	3
TAOK3	10	-2
TBK1	1	-2
TBK1	10	-7
TEC	1	2
TEC	10	1
TIE2	1	0
TIE2	10	-9
TNIK	1	1
TNIK	10	1
TNK2	1	3
TNK2	10	2
TRKA	1	2
TRKA	10	-5
TRKB	1	5
TRKB	10	-3
TRKC	1	3
TRKC	10	-3

Kinase	Conc. Tested (μM)	CP2
TSSK1	1	-2
TSSK1	10	7
TSSK2	1	-2
TSSK2	10	1
TTK	1	2
TTK	10	4
TXK	1	6
TXK	10	-5
TYK2	1	4
TYK2	10	-7
TYRO3	1	0
TYRO3	10	-4
YES	1	-1
YES	10	-4
ZAP70	1	-15
ZAP70	10	-3

Supplementary Table 3. Results of the Eurofin Cerep Safety-Screen 44 Panel (enzymes)

Assay	Test Concentration (M)	% Inhibition of Control Values
COX1 (h)	1.0E-05	17
COX2 (h)	1.0E-05	17
PDE3A (h)	1.0E-05	13
PDE4D2 (h)	1.0E-05	28
Lck kinase (h)	1.0E-05	-5
acetylcholinesterase (h)	1.0E-05	33

In vitro pharmacological profiling and assessment of the potential for off-target interactions of CP2 in binding screens (Eurofins Cerep-Panlabs SafetyScreen 44). The compound was screened at a 10 μM concentration in duplicate for its potential to interfere with the binding of native ligands of 44 different receptors, ion channels and enzymes. For enzyme assays, the inhibition effect was calculated as percent inhibition of control enzyme activity. Tests were performed in duplicate and results are averages of those two tests. Results showing an inhibition (or stimulation for assays run in basal conditions) higher than 50% are considered to represent significant effect of the test compound. Results showing an inhibition (or stimulation) between 25% and 50% are indicative of weak to moderate effect. Results showing an inhibition (or stimulation) lower than 25% are not considered significant and mostly attributable to variability of the signal around the control level.

Supplementary Table 4. Results of the Eurofin Cerep Safety-Screen 44 Panel (receptors and ion channels)[a]

Assay	Test concentration (M)	% Inhibition of Control Specific Binding
A2A (h) (agonist radioligand)	1.0E-05	15
alpha 1A (h) (antagonist radioligand)	1.0E-05	12
alpha 2A (h) (antagonist radioligand)	1.0E-05	-7
beta 1 (h) (agonist radioligand)	1.0E-05	5
beta 2 (h) (agonist radioligand)	1.0E-05	-1
BZD (central) (agonist radioligand)	1.0E-05	11
CB1 (h) (agonist radioligand)	1.0E-05	-14
CB2 (h) (agonist radioligand)	1.0E-05	-6
CCK1 (CCKA) (h) (agonist radioligand)	1.0E-05	-3
D1 (h) (antagonist radioligand)	1.0E-05	-9
D2S (h) (agonist radioligand)	1.0E-05	0
ETA (h) (agonist radioligand)	1.0E-05	-1
NMDA (antagonist radioligand)	1.0E-05	3
H1 (h) (antagonist radioligand)	1.0E-05	8
H2 (h) (antagonist radioligand)	1.0E-05	-3
MAO-A (antagonist radioligand)	1.0E-05	25
M1 (h) (antagonist radioligand)	1.0E-05	35
M2 (h) (antagonist radioligand)	1.0E-05	50
M3 (h) (antagonist radioligand)	1.0E-05	30
N neuronal alpha 4beta 2 (h) (agonist radioligand)	1.0E-05	5
delta 2 (DOP) (h) (agonist radioligand)	1.0E-05	26
kappa (KOP) (agonist radioligand)	1.0E-05	3
mu (MOP) (h) (agonist radioligand)	1.0E-05	28
5-HT1A (h) (agonist radioligand)	1.0E-05	0
5-HT1B (antagonist radioligand)	1.0E-05	-12
5-HT2A (h) (agonist radioligand)	1.0E-05	-2
5-HT2B (h) (agonist radioligand)	1.0E-05	1
5-HT3 (h) (antagonist radioligand)	1.0E-05	0
GR (h) (agonist radioligand)	1.0E-05	3
AR (h) (agonist radioligand)	1.0E-05	18
V1a (h) (agonist radioligand)	1.0E-05	10
Ca2+ channel (L, dihydropyridine site) (antagonist radioligand)	1.0E-05	8
Potassium Channel hERG (human), [3H] Dofetilide	1.0E-05	71
KV channel (antagonist radioligand)	1.0E-05	-4
Na+ channel (site 2) (antagonist radioligand)	1.0E-05	34
norepinephrine transporter (h) (antagonist radioligand)	1.0E-05	8
dopamine transporter (h) (antagonist radioligand)	1.0E-05	32
5-HT transporter (h) (antagonist radioligand)	1.0E-05	0

[a]In vitro pharmacological profiling and assessment of the potential for off-target interactions of CP2 in binding screens (Eurofins Cerep-Panlabs SafetyScreen 44). The compound was screened at a 10 µM concentration in duplicate for its potential to interfere with the binding of native ligands of 44 different receptors, ion channels and enzymes. For receptor assays, compound binding was calculated as percent inhibition of the binding of a radioactively labeled ligand specific for each target. Tests were performed in duplicate and results are averages of those two tests. Results showing an inhibition (or stimulation for assays run in basal conditions) higher than 50% are considered to represent significant effect of the test compound. Results showing an inhibition (or stimulation) between 25% and 50% are indicative of weak to moderate effect. Results showing an inhibition (or stimulation) lower than 25% are not considered significant and mostly attributable to variability of the signal around the control level.

Supplementary Table 5. Effect of CP2 treatment on brain metabolite levels in APP/PS1 and NTG mice[a].

	APP/PS1		APP/PS1 (CP2)		t-test APP/PS1-CP2	NTG		NTG(CP2)		t-test NTG-CP2
BRAIN	$\bar{X}$	± SE	$\bar{X}$	± SE	P values	$\bar{X}$	± SE	$\bar{X}$	± SE	P values
2-Hydroxyglutarate	1.28	0.10	1.19	0.11	0.307	0.76	0.09	0.93	0.20	0.265
γ-Aminobutyrate	0.89	0.05	1.07	0.07	0.062	0.98	0.15	0.96	0.04	0.448
Adenosine	1.25	0.09	0.97	0.16	0.119	0.96	0.18	0.76	0.06	0.195
ADP	0.89	0.08	1.08	0.06	0.078	1.03	0.09	1.01	0.08	0.458
Alanine	0.94	0.02	1.27	0.11	***0.033***	0.96	0.07	0.94	0.07	0.433
AMP	0.97	0.04	1.20	0.02	***0.003***	0.89	0.04	0.91	0.05	0.395
Ascorbate	0.95	0.14	1.59	0.22	***0.039***	0.76	0.18	0.74	0.20	0.472
Aspartate	0.52	0.05	0.90	0.22	0.107	0.94	0.18	1.35	0.16	0.088
ATP	0.78	0.15	1.15	0.23	0.140	1.10	0.18	1.02	0.14	0.387
β-Alanine	0.30	0.11	1.11	0.08	***0.001***	0.88	0.22	0.86	0.10	0.469
Cholestrol	0.84	0.20	0.69	0.29	0.359	1.18	0.38	1.46	0.51	0.351
Citrate	0.91	0.04	1.04	0.01	***0.029***	1.07	0.04	0.98	0.09	0.238
Creatinine	1.02	0.11	1.13	0.03	0.216	1.04	0.08	0.81	0.08	0.058
Dehydroascorbate	0.70	0.06	1.61	0.26	***0.025***	0.83	0.17	1.01	0.21	0.294
Ethanolamine	0.89	0.16	1.17	0.07	0.118	1.07	0.11	1.13	0.07	0.333
Fumarate	0.67	0.32	1.13	0.26	0.181	0.56	0.23	1.17	0.21	0.064
Galactose	0.95	0.12	1.03	0.24	0.395	0.84	0.09	1.15	0.18	0.123
GDP	0.84	0.04	1.13	0.03	***0.002***	0.92	0.13	1.04	0.05	0.237
Glucopyranose	1.56	0.17	1.08	0.13	***0.045***	0.89	0.13	0.42	0.06	***0.019***
Glutamate	0.99	0.09	1.15	0.15	0.210	1.05	0.10	0.91	0.25	0.337
Glycerate	1.03	0.15	0.78	0.06	0.117	1.19	0.09	0.74	0.06	***0.005***
Glycerol -1-P	1.00	0.05	1.05	0.06	0.267	1.00	0.11	1.02	0.10	0.461
Glycine	0.69	0.04	0.88	0.08	0.053	0.97	0.10	1.10	0.03	0.159
Glycolate	0.56	0.21	0.17	0.00	0.094	0.69	0.12	1.00	0.12	0.081
GTP	0.88	0.05	1.19	0.07	***0.012***	0.98	0.07	0.96	0.08	0.441
Iminodiacetate	1.13	0.08	0.86	0.21	0.172	0.91	0.24	0.55	0.14	0.153
Lactate	1.10	0.03	1.04	0.01	0.057	0.93	0.06	0.95	0.01	0.393
Malate	0.96	0.09	0.98	0.07	0.448	1.02	0.02	1.09	0.11	0.319
Myo-Inositol	0.91	0.03	1.04	0.06	0.072	1.00	0.05	0.98	0.03	0.364
NAA	0.94	0.02	1.10	0.03	***0.004***	1.00	0.06	1.04	0.09	0.369
Phosphocolamine	0.84	0.07	1.16	0.13	0.061	0.90	0.07	1.11	0.05	***0.036***
Pi	0.86	0.07	0.98	0.02	0.119	1.00	0.04	1.07	0.04	0.152
Pyroglutamate	0.52	0.07	0.84	0.07	***0.011***	0.94	0.15	1.28	0.07	0.070
Serine	0.65	0.05	0.97	0.05	***0.003***	0.91	0.10	1.20	0.09	***0.050***
Stearate	1.03	0.04	0.93	0.05	0.111	0.96	0.16	1.15	0.14	0.225
Succinate	1.08	0.10	1.07	0.13	0.489	0.98	0.06	0.91	0.13	0.352
Threonate	0.89	0.07	1.03	0.14	0.225	1.17	0.10	1.10	0.13	0.375
Urea	1.04	0.08	1.20	0.03	0.092	0.94	0.08	0.71	0.04	***0.037***
Valine	0.47	0.11	0.96	0.10	***0.013***	0.79	0.14	0.43	0.08	***0.050***

[a]Metabolomics was conducted in mice treated with CP2 or vehicle for 6 months. 5 mice were included in each group. Data were analyzed by unpaired Student *t*-test. P < 0.05 was considered significant.

Bold italic indicates significant changes
